# A biofilm-targeting lipo-peptoid to treat *Pseudomonas aeruginosa* and *Staphylococcus aureus* co-infections

**DOI:** 10.1016/j.bioflm.2025.100272

**Published:** 2025-03-12

**Authors:** Samuel J.T. Wardell, Deborah B.Y. Yung, Josefine E. Nielsen, Rajesh Lamichhane, Kristian Sørensen, Natalia Molchanova, Claudine Herlan, Jennifer S. Lin, Stefan Bräse, Lyn M. Wise, Annelise E. Barron, Daniel Pletzer

**Affiliations:** a Department of Microbiology and Immunology, University of Otago, Dunedin, New Zealand; b Maurice Wilkins Centre for Molecular Biodiscovery, The University of Auckland, Auckland, 1042, New Zealand; c Department of Bioengineering, Stanford University, School of Medicine, Stanford, CA 94305, USA; d Department of Science and Environment, Roskilde University, 4000, Roskilde, Denmark; e The Molecular Foundry, Lawrence Berkeley National Laboratory, Berkeley, CA, 94720, USA; f Institute of Biological and Chemical Systems - Functional Molecular Systems (IBCS-FMS), Karlsruhe Institute of Technology (KIT), Germany; g Department of Pharmacology and Toxicology, University of Otago, Dunedin, New Zealand

**Keywords:** Polymicrobial, Host-mimicking conditions, Abscess model, Biofilms, Peptides, Peptoids

## Abstract

Antibiotic-resistant bacterial infections are a significant clinical challenge, especially when involving multiple species. Antimicrobial peptides and their synthetic analogues, peptoids, which target bacterial cell membranes as well as intracellular components, offer potential solutions. We evaluated the biological activities of novel peptoids TM11-TM20, which include an additional charged *N*Lys residue, against multidrug-resistant *Pseudomonas aeruginosa* and *Staphylococcus aureus*, both *in vitro* and *in vivo*. Building on insights from previously reported compounds TM1-TM10, the lipo-peptoid TM18, which forms self-assembled ellipsoidal micelles, demonstrated potent antimicrobial, anti-biofilm, and anti-abscess activity. Transcriptome sequencing (RNA-seq) revealed that TM18 disrupted gene expression pathways linked to antibiotic resistance and tolerance, and biofilm formation in both pathogens. Under dual-species conditions, TM18 induced overlapping but attenuated transcriptional changes, suggesting a priming effect that enhances bacterial tolerance. In a murine skin infection model, TM18 significantly reduced dermonecrosis and bacterial burden in mono-species infections. When combined with the antibiotic meropenem, they synergistically nearly cleared co-infections. Our findings highlight that TM18 has potential as a novel therapeutic for combating antibiotic-resistant pathogens and associated biofilm-driven tolerance.

## Highlights

• Lipo-peptoid TM18 disrupts biofilms formed by antibiotic-resistant pathogens.
• TM18 self-assembles into core-shell ellipsoidal micelles with potent antimicrobial activity.
• TM18 modulates bacterial gene expression, impacting biofilm tolerance mechanisms.
• TM18 shows strong anti-abscess effects in a murine skin infection model.
• TM18 synergizes with meropenem, significantly reducing polymicrobial infections *in vivo*.

## Significance statement

Antibiotic-resistant infections are increasingly complex, especially when multiple bacterial species are involved. This study introduces a novel lipo-peptoid, TM18, as a potent antimicrobial agent that effectively disrupts the defenses of multidrug-resistant *Pseudomonas aeruginosa* and *Staphylococcus aureus*, both individually and in dual-species settings. By targeting both biofilm formation and gene expression pathways related to bacterial tolerance, TM18 not only reduces bacterial load but also alleviates dermonecrosis in a murine infection model. Notably, the combined use of TM18 and meropenem approaches near eradication of co-infections. This work highlights TM18's potential to fill critical gaps in the treatment of dual-species infections and improve clinical outcomes against challenging antibiotic-resistant pathogens.

## 1 Introduction

Chronic infections are a significant burden worldwide, with approximately 80% attributed to biofilms [1]. Biofilms are aggregates of bacteria encased in a self-produced extracellular matrix (ECM) that protects them from the environment and allows them to adhere to various surfaces [2,3]. Bacteria within biofilms are challenging to treat due to structural and phenotypic heterogeneity, which includes limited antimicrobial penetration of the ECM, but also physiological adaptations of biofilm-associated cells, including metabolic dormancy of some bacterial subpopulations, protection from the host immune system, negatively charged extracellular DNA decreasing antibiotic efficacy, increased horizontal gene transfer of resistance genes, and complex cell-to-cell interactions that enable synergistic cooperation [[3], [4], [5]]. Biofilms are frequently polymicrobial, involving two or more species, and are often associated with worse patient outcomes [6]. *Pseudomonas aeruginosa* and *Staphylococcus aureus* are commonly co-isolated from chronic infections such as otitis media, chronic wound infections, and lung infections in cystic fibrosis patients [7]. The complex nature of biofilms often renders antibiotic therapy ineffective, allowing infections to persist.

The importance of polymicrobial infections and biofilm-associated infections has been increasingly recognized [8,9], highlighting the need for effective treatment strategies. Different bacterial species can co-evolve and interact during these infections, leading to increased tolerance and antibiotic resistance [10,11]. Current methods of studying multispecies polymicrobial infections and biofilms are limited as *in vitro* co-culture techniques often favor one species over others. Additionally, resistance quantification typically uses standard antimicrobial susceptibility tests and antibiofilm activity assays in laboratory media, which do not capture aspects of the complex host environment and bacterial interactions [12,13]. Indeed, it is well-established that the host environment has a major impact on the efficacy of antimicrobial compounds against pathogens, influencing and interfering with compound activity [14].

Antimicrobial peptides (AMPs) have emerged as a promising avenue to combat biofilm-associated infections due to their broad-spectrum activity against both Gram-negative and Gram-positive bacteria, parasites, and viruses [15,16] and activity in both standard laboratory media and host-mimicking conditions [17]. However, many peptides exhibit drawbacks such as poor stability, susceptibility to proteolytic degradation, and poor absorption [18]. To overcome these limitations, peptidomimetics, which are more stable than peptides, resistant to enzymatic degradation, and have higher selectivity, have been developed and studied [19]. Peptidomimetics include peptoids, synthetic molecules that mimic peptide structures but are less susceptible to enzymatic degradation and show excellent *in vivo* stability due to the amino acid functional side chain being appended to the nitrogen atom rather than to the α-carbon in the oligoamide backbone [20]. Antimicrobial peptoids self-assemble into complex multimeric structures, including helical bundles, core-shell ellipsoidal micelles, or worm-like micelles, with the most efficacious compounds forming monodisperse ellipsoidal micelles in particular [17,21,22]. Critically, peptoids have demonstrated strong activity against biofilm-associated bacteria [17,23,24].

Previously, we showed that peptoids TM1-TM10 exhibit antimicrobial and antibiofilm activity against mono- and dual-species biofilms *in vitro*, as well as anti-abscess activity against mono-species infections of *P. aeruginosa* and *S. aureus* [17]. The bioactivity of these peptoids was associated with their supramolecular assembly. Here we introduce new peptoids TM11-TM20, variants of TM1-TM10 with an added *N*Lys group to increase the net positive charge. We demonstrate that lipo-peptoid TM18 exhibited potent activity against co-infections of *P. aeruginosa* and *S. aureus in vitro* and *in vivo* and synergized with the carbapenem antibiotic meropenem *in vivo*.

## 2 Results

### 2.1 Peptoids maintained their antimicrobial activity against *P. aeruginosa* and *S. aureus* under conditions that mimic the host environment

The host environment is composed of various components that can affect drug effectiveness [25,26]. In our previous study, we demonstrated the broad-spectrum activity of peptoids TM1-TM10 against all *ESKAPE* pathogens. We also found that the antimicrobial activity of some peptoids was altered when tested against *P. aeruginosa* and *S. aureus* under host-mimicking conditions [17]. Here, we screened a new library of ten peptoids (TM11-TM20), which are variants of our previously published peptoids with an added C-terminal *N*Lys residue, introducing an additional positive charge. Specifically, TM11 is TM1 with an additional *N*Lys, TM12 is TM2 + *N*Lys, etc. This single *N*Lys monomer has been shown to increase selectivity for bacteria over mammalian cells without affecting potency [27].

We selected the multidrug-resistant Liverpool Epidemic strain *P. aeruginosa* B58 (LESB58) and methicillin-resistant *S. aureus* (MRSA) USA300 LAC as model organisms to test the new peptoid library under standard laboratory conditions (Mueller Hinton Broth; MHB) and physiologically relevant conditions (tissue culture medium supplemented with fetal bovine serum and glucose, DFG) (Table 1). An inactive control peptoid lacking aromatic side chains [28], TM22 [29], was included as well.

**Table 1:** MIC (μg/mL) of peptoids against multidrug-resistant *P. aeruginosa* LESB58 and methicillin-resistant *S. aureus* USA300 LAC in MHB and DFG.

	TM 11	TM 12	TM 13	TM 14	TM 15	TM 16	TM 17	TM 18	TM 19	TM 20	TM 22
*P. aeruginosa* LESB58											
DFG	50	100	100	25	25	12.5	>100	12.5	25	25	>100
MHB	50	>100	100	25	100	100	>100	6.3	12.5	25	>100
*S. aureus* LAC USA300											
DFG	1.6	1.6	3.1	1.6	1.6	0.6	12.5	1.6	3.1	6.3	>100
MHB	6.3	6.3	6.3	6.3	3.1	0.6	25	6.3	12.5	12.5	>100

The MICs against *P. aeruginosa* ranged from 6.25 to >100 μg/mL, with half of the peptoids showing no change in activity under host-mimicking conditions. Interestingly, TM15 and TM16 exhibited a 4- and 8-fold increase in activity, respectively, in DFG. In contrast, TM18 and TM19 showed a 2-fold decrease in activity in DFG against *P. aeruginosa*. Despite the decrease, TM18 exhibited the most potent activity against *P. aeruginosa* (12.5 μg/mL) under host-mimicking conditions. TM16 also demonstrated strong activity against *P. aeruginosa* in DFG and had the best activity against *S. aureus* (0.63 μg/mL).

On the other hand, all peptoids, except TM22, demonstrated strong activity against *S. aureus* with MIC values ranging from 0.63 to 12.5 μg/mL in both media. Most peptoids, except TM16, showed a 2-4-fold increase in activity against *S. aureus* under host-mimicking conditions compared to standard laboratory conditions.

### 2.2 Peptoids eradicated *P. aeruginosa*-*S. aureus* dual-species biofilms

Peptoids have emerged as promising agents with significant antibiofilm activity against both mono- and dual-species biofilms [17,23]. In our previous studies, we demonstrated that TM1-TM10 exhibited excellent antibiofilm activity, effectively killing bacteria within biofilms [17]. Here, we further investigated the efficacy of the new variants TM11-TM20 against biofilms formed by *P. aeruginosa* and *S. aureus*, both individually and in combination. Like the original peptoids [17], the new variants also did not exhibit strong activity against *P. aeruginosa* biomass removal as measured by crystal violet staining. However, many of the new peptoids were highly effective in reducing the biofilm mass of *S. aureus* by more than 50 % (Figs. S1A and S1B).

**Fig. 1:**
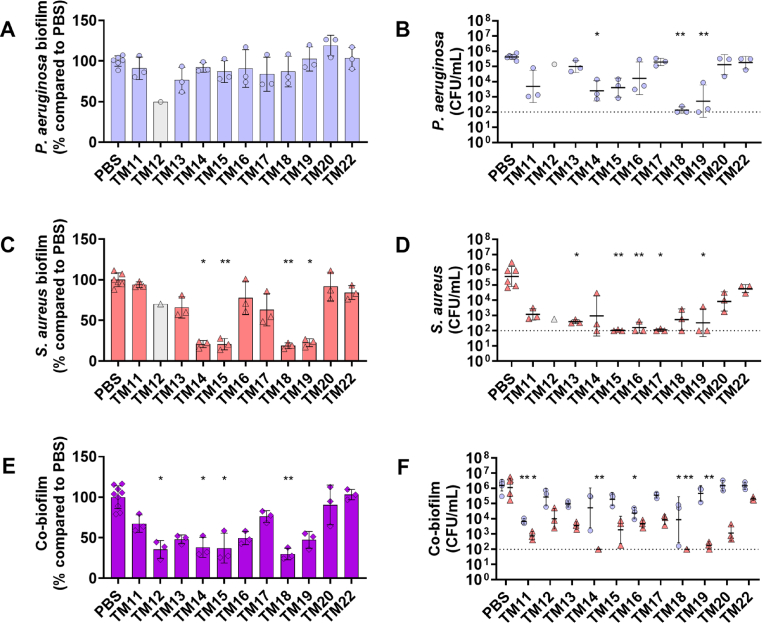
**Antibiofilm activities of peptoids against mono-species and dual-species biofilms.** Peptoids (31.25 μg/mL) were used to treat mono-species biofilms of (**A**, **B**) *P. aeruginosa* (purple circles), and (**C**, **D**) *S. aureus* (red triangles)*,* as well as (**E, F**) dual-species biofilms of *P. aeruginosa*-*S. aureus*. Biofilms were grown for 20–24 h in DFG in 96-well plates before peptoid treatment, followed by re-incubation for 24 h. (**A**, **C, E**) Biofilm mass was quantified by staining with 0.1 % crystal violet and (**B**, **D**, **F**) bacterial survivors were determined on selective agar plates. TM12 data (grey circle/triangle; n = 1) was excluded from statistical analysis due to limited sample availability. Significance levels are indicated as ∗*p* < 0.05 and ∗∗*p* < 0.01 based on the Kruskal−Wallis test with Dunn's correction. Dashed line represents the limit of detection. (For interpretation of the references to color in this figure legend, the reader is referred to the Web version of this article.)

To gain further insights into their killing activity within biofilms, we used a fixed peptoid concentration of 31.25 μg/mL, determined by the overall antibiofilm activity of all peptoids and our previous studies [17]. This concentration is approximately at the *P. aeruginosa* MIC and 10–50 times the *S. aureus* MIC for most peptoids. As expected, peptoids at 31.25 μg/mL did not reduce mono-species *P. aeruginosa* biofilm mass (Fig. 1A). However, TM14, TM18, and TM19 significantly reduced bacterial survivors within the biofilm (Fig. 1B). In contrast, peptoids TM14, TM15, TM18, and TM19 exhibited a significant reduction of *S. aureus* biofilm mass (Fig. 1C), while TM15, TM16, TM17, and TM19 also significantly reduced *S. aureus* survivors within mono-species biofilms (Fig. 1D).

We also previously used this specific peptoid concentration to treat *P. aeruginosa-S. aureus* dual-species biofilms [17]. Therefore, we further investigated the biofilm eradication activity against these biofilms. We found that TM12, TM14, TM15, and TM18 significantly reduced dual-species biofilm mass by more than 50% compared to untreated biofilms (Fig. 1E). Intriguingly, peptoids TM11, TM16 and TM18 displayed enhanced killing against *P. aeruginosa,* reducing bacterial survivors by approximately 1000-, 100-, and 100-fold, respectively. Peptoids TM12, TM13, TM15, TM16, TM17, TM20, and the control peptoid TM22 showed no significant reduction in *S. aureus* in dual-species biofilms (Fig. 1F). The most promising candidates, TM11, TM14, TM18 and TM19, reduced *S. aureus* bacterial survivors by 1000- to 10,000-fold.

Due to peptoid TM18 showing the greatest promise as an effective molecule, it was tested against additional *P. aeruginosa* (PAO1 and PA14) and *S. aureus* (HG001 and Newman) strains, showing broad-spectrum antimicrobial (Table S8) and antibiofilm activity (Fig. S2).

### 2.3 Lipo-peptoid TM18 self-assembled into core-shell ellipsoidal micelles

**Fig. 2:**
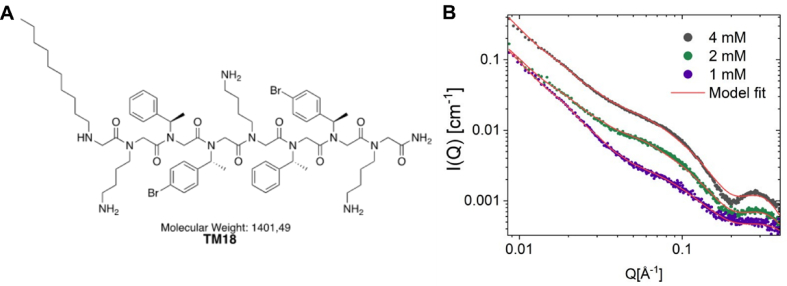
**Structural and SAXS analysis of TM18.** A) Chemical structure of TM18. B) SAXS data for TM18 measured at 1 mM, 2 mM, and 4 mM plotted together with the best fit (red line) using an ellipsoidal core shell-micellar model described in [17]. (For interpretation of the references to color in this figure legend, the reader is referred to the Web version of this article.)

Among the peptoids screened for antimicrobial and anti-biofilm activity, the lipo-peptoid TM18 emerged as a particularly promising candidate due to its broad-spectrum antimicrobial activity and capability to reduce bacterial survivors within biofilms. Lipo-peptoids combine the structural features of peptides and lipids, enhancing their ability to interact with and disrupt bacterial membranes. To characterize the self-assembled structure of TM18 (H-*N*dec-(*N*Lys-*N*spe-*N*spe)_2_-*N*Lys-NH_2_) in aqueous solution, we conducted synchrotron SAXS (Small-Angle X-ray Scattering) experiments and analyzed the data using theoretical modelling (Fig. 2). The results revealed that TM18 self-assembles into core-shell ellipsoidal micelles, similar to those shown for TM5 and TM8, which are well-characterized peptoids with potent broad-spectrum antimicrobial activity [17,30].

However, TM18 and TM8 have a slightly thicker shell (9 Å compared to 7 Å for TM5), which can be attributed to the two and three extra residues in the peptoid sequence. Similar to TM8, TM18 has a slightly smaller core than TM5 (10 Å compared to 13 Å for TM5), likely due to the shorter alkyl tail (*N*dec rather than *N*tridec). The scattering also indicated the presence of a small fraction of larger aggregates (0.08), evidenced by a sharp upturn at low Q (∼0.009−0.03), similarly as observed for TM8 [17].

### 2.4 TM18 induced transcriptional tolerance in *P. aeruginosa* and metabolic changes in *S. aureus* biofilms

Given the strong broad-spectrum, anti-biofilm activity of TM18 against mono- and dual-species *P. aeruginosa* and *S. aureus* biofilms, we further investigated the transcriptional regulatory responses using RNA-seq. Here, we utilized a static biofilm model on hydroxyapatite discs, which more accurately mimics biological surfaces and supports biofilm maturation [31,32]. Mono- and dual-species biofilms were grown under host-mimicking conditions and treated with TM18 for 1 h to capture an early treatment response. There was a considerable overlap (∼30 %) in the genes significantly dysregulated in response to TM18 treatment in both mono- and dual-species biofilms (Fig. S3).

For *P. aeruginosa* mono-species biofilms, TM18 treatment led to modest transcriptional changes with 96 genes significantly differentially expressed (FDR adjusted *p-*value <0.05 and fold-change >1.5 or < -1.5; 59 genes upregulated, 37 genes downregulated), likely due to the short treatment duration and its ability to tolerate the treatment, at least for a short period. Notably, TM18 treatment resulted in widespread upregulation of RND (resistance-nodulation-cell division)-type efflux systems, including *mexCD-oprJ*, *mexEF-oprN mexJK*, *mexGHI-opmD* and *muxABC-opmB* (Table 2, Table S1, Fig. S4). Additionally, the PmrAB two-component system, which modulates resistance to cationic antimicrobial peptides [33], was upregulated ∼20 fold, along with LPS aminoarabinosylation lipid A modification system, *arn* [34] (Table 2, Fig. S4). Interestingly, the outer membrane porin *oprD* was significantly dysregulated, showing a >3-fold downregulation when treated with TM18 in mono-species biofilms. Phenazine biosynthesis was also highly upregulated upon TM18 treatment (Fig. S4).

**Table 2:** **Differentially expressed genes for *P. aeruginosa* mono-species biofilms treated with 32 μg/mL TM18, compared to dual-species biofilms.** Fold change (FC) relative to untreated samples, with positive values indicating increased expression when treated with TM18.

Loci	Gene	Description	FC mono-species biofilms	FC dual-species biofilms
***Quorum sensing***				
PALES_43251	*pqsE*	Quinolone signal response protein	4.4	1.5
PALES_43261	*pqsD*	3-oxoacyl-(acyl carrier protein) synthase III	4.0	1.4
PALES_43271	*pqsC*	beta-keto-acyl-acyl-carrier protein synthase	3.0	1.2
PALES_43281	*pqsB*	Homologous to beta-keto-acyl-acyl-carrier protein synthase	3.2	1.5
PALES_43291	*pqsA*	coenzyme A ligase	2.7	1.4

***Efflux/uptake systems***				
PALES_28031	*mexE*	Resistance-Nodulation-Cell Division (RND) multidrug efflux membrane fusion protein	1.6	1.2
PALES_27691	*opmB*	Outer membrane protein precursor	3.6	2.6^a^
PALES_27681	*muxC*	(RND) efflux transporter	4.2	2.6^a^
PALES_27671	*muxB*	(RND) efflux transporter	4.4	2.3^a^
PALES_27661	*muxA*	(RND) efflux membrane fusion protein	3.1	2.2^a^
PALES_13081	*mexK*	(RND) efflux transporter	2.7	1.8^a^
PALES_13071	*mexJ*	(RND) efflux membrane fusion protein	2.7	1.0
PALES_07201	*mexI*	(RND) efflux transporter	2.0	1.6
PALES_49821	*oprJ*	Multidrug efflux outer membrane protein	10.8	24.0^a^
PALES_49831	*mexD*	(RND) multidrug efflux transporter	10.3	12.0^a^
PALES_49841	*mexC*	(RND) multidrug efflux membrane fusion protein	4.1	9.3^a^
PALES_43561	*oprD*	Basic amino acid, basic peptide and imipenem outer membrane porin	−3.2	−1.2

***Outer membrane/LPS***				
PALES_20851	*murB*	UDP-N-acetylenolpyruvoylglucosamine reductase	1.6	−1.0
PALES_14811	*arnB*	UDP-4-amino-4-deoxy-l-arabinose-oxoglutarate aminotransferase	29.4	16.7^a^
PALES_14801	*arnC*	Glycosyl transferase	45.5	19.7^a^
PALES_14791	*arnA*	Bifunctional UDP-glucuronic acid decarboxylase/UDP-4-amino-4-deoxy-l-arabinose formyltransferase	45.1	16.8^a^
PALES_14781	*arnD*	Olysaccharide deacetylase	31.6	20.8^a^
PALES_14771	*arnT*	4-amino-4-deoxy-l-arabinose transferase	92.1	8.8^a^
PALES_14761	*arnE*	Inner membrane protein	16.4	14.3^a^
PALES_14751	*arnF*	Hypothetical protein	92.3	38.3^a^
PALES_14741	PALES_14741	Nucleotide sugar dehydrogenase	51.3	17.0^a^
PALES_50471	*pagL*	Lipid A 3-*O*-deacylase	2.6	2.3^a^
PALES_51611	*pmrA*	Two-component regulator system response regulator	21.3	14.3^a^
PALES_51621	*pmrB*	Two-component regulator system signal sensor kinase	17.8	16.2^a^

***Periplasm/PMF***				
PALES_41491	*napC*	Cytochrome c-type protein	−2.0	1.3
PALES_41481	*napB*	Cytochrome c-type protein precursor	−1.5	1.3
PALES_41471	*napA*	Periplasmic nitrate reductase protein	−1.4	−1.1
PALES_41461	*napD*	Protein of periplasmic nitrate reductase	−2.8	1.5
PALES_41451	*napE*	Periplasmic nitrate reductase protein	−1.5	−1.2
PALES_37731	*ccoP2*	Cytochrome *c* oxidase, cbb3-type, CcoP	−2.3	1.6
PALES_37721	*ccoO2*	Cytochrome *c* oxidase, cbb3-type, CcoO	−2.6	1.4
PALES_37711	*ccoN2*	Cytochrome *c* oxidase, cbb3-type, CcoN	−1.9	1.0
PALES_49541	PALES_49541	Putative cytochrome *c*	−2.0	−1.0

a Denotes genes significantly differentially expressed in dual-species biofilms. All genes listed were significantly differentially expressed in mono-microbial biofilms.

When examining *P. aeruginosa* response to TM18 under dual-species biofilm conditions, the transcriptional response was less substantial, with only 48 genes significantly differentially expressed (Table S4, Fig. S5). Although there was a large overlap between the mono-species and dual-species response (Fig. S3), many key genes were no longer significantly differentially expressed (Table 2). Comparing the *P. aeruginosa* gene expression under dual-species conditions with *S. aureus* to mono-species conditions, it was evident that the presence of *S. aureus* primed *P. aeruginosa* by upregulating specific genes. This priming effect resulted in less pronounced dysregulation of gene expression but increased tolerance to environmental stressors and antimicrobial agents in dual-species environments. Specifically, these priming mechanisms included a strong upregulation of the aminoarabinose biosynthesis pathway, various efflux pump systems and the expression of porin proteins.

In contrast, treatment of *S. aureus* mono-species biofilms with TM18 caused the dysregulation of 665 genes (283 genes upregulated, 362 genes downregulated). This response reflects a more pronounced impact on *S. aureus* compared to *P. aeruginosa,* likely due to the high peptoid concentration, which may have induced cell death. The observed transcriptional changes included dysregulation in the expression of proteases and peptidases, with >2-fold downregulation of serine proteases and lysins, and >2-fold downregulation of the type VII secretion system. Additionally, genes associated with phosphate metabolism were >2-fold upregulated (Table 3). There was also a significant upregulation of genes involved in fatty acid synthesis and nicotinamide adenine dinucleotide (NAD) binding, which are crucial for redox reactions. Metabolic pathways, particularly those related to amino acid metabolism, were also significantly upregulated. Conversely, genes associated with adhesion and cell wall synthesis were predominantly downregulated (Fig. S6).

**Table 3:** **Differentially expressed genes for *S. aureus* mono-species biofilms treated with 32 μg/mL TM18, compared to dual-species biofilms.** Fold change (FC) relative to untreated samples, with positive values indicating increased expression when treated with TM18.

Loci	Gene	Description	FC mono-species biofilms	FC dual-species biofilms
***Protease/peptidase***				
SAUSA300_1146	*hslV*	ATP-dependent protease HslV	2.0	1.4
SAUSA300_1147	*hslU*	ATP-dependent protease ATPase HslU	1.7	1.3
SAUSA300_1402	SAUSA300_1402	Clp protease ClpP	−1.6	1.7
SAUSA300_1753	*splF*	Serine protease SplF	−2.5	1.3
SAUSA300_1754	*splE*	Serine protease SplE	−2.5	1.1
SAUSA300_1755	*splD*	Serine protease SplD	−2.5	−1.0
SAUSA300_1756	*splC*	Serine protease SplC	−2.5	−2.0
SAUSA300_1757	*splB*	Serine protease SplB	−3.7	−1.0
SAUSA300_1758	*splA*	Serine protease SplA	−3.5	−2.3
SAUSA300_1890	*scpA*	Cysteine protease staphopain A	−1.9	−1.3
SAUSA300_0207	SAUSA300_0207	M23 family metallopeptidase	5.7	4.3^a^
SAUSA300_0752	*clpP*	ATP-dependent Clp endopeptidase proteolytic subunit	1.6	1.4
SAUSA300_1351	SAUSA300_1351	Zinc metallopeptidase	1.6	2.1^a^
SAUSA300_1674	SAUSA300_1674	Trypsin-like peptidase domain-containing protein	2.0	1.1
SAUSA300_1691	SAUSA300_1691	M42 family metallopeptidase	1.6	2.3^a^
SAUSA300_2381	SAUSA300_2381	C39 family peptidase	2.3	−1.4
SAUSA300_2400	SAUSA300_2400	M42 family metallopeptidase	1.6	1.1

***Phosphate metabolism***				
SAUSA300_0145	SAUSA300_0145	Phosphate/phosphite/phosphonate ABC transporter substrate-binding protein	1.5	−1.1
SAUSA300_0216	*uhpT*	Hexose-6-phosphate:phosphate antiporter	−4.0	−2.6^a^
SAUSA300_0360	SAUSA300_0360	Aminotransferase class I/II-fold pyridoxal phosphate-dependent enzyme	2.0	1.5
SAUSA300_1122	*plsX*	Phosphate acyltransferase	2.1	−1.0
SAUSA300_1279	*phoU*	Phosphate signaling complex protein	2.6	2.5^a^
SAUSA300_1280	SAUSA300_1280	Phosphate ABC transporter ATP-binding protein	2.6	2.3^a^
SAUSA300_1281	*pstA*	Phosphate ABC transporter permease	2.8	2.3^a^
SAUSA300_1282	*pstC*	Phosphate ABC transporter permease	2.6	2.1^a^
SAUSA300_1283	SAUSA300_1283	Phosphate ABC transporter substrate-binding protein	2.6	2.3^a^

***Type VII secretion system***				
SAUSA300_0278	*esxA*	WXG100 family type VII secretion effector EsxA	−2.0	1.5
SAUSA300_0279	*esaA*	Type VII secretion protein EsaA	−3.0	−2.6^a^
SAUSA300_0280	*essA*	Type VII secretion protein EssA	−3.0	−1.9^a^
SAUSA300_0281	*esaB*	Type VII secretion protein EsaB	−1.9	−1.7
SAUSA300_0282	*essB*	Type VII secretion protein EssB	−3.0	−2.0^a^
SAUSA300_0283	*essC*	Type VII secretion protein EssC	−2.5	−1.5
SAUSA300_0284	*esaC*	Type VII secretion substrate EsaC	−2.5	−1.3
SAUSA300_0285	*esxB*	WXG100 family type VII secretion effector EsxB	−2.0	−1.3
SAUSA300_2524	SAUSA300_2524	TIGR04197 family type VII secretion effector	−1.6	−2.0^a^

***Lysins***				
SAUSA300_2365	*hlgA*	Bi-component gamma-hemolysin HlgAB subunit A	−13.0	−16.0^a^
SAUSA300_2366	*hlgC*	Bi-component gamma-hemolysin HlgCB subunit C	−9.8	−17.1^a^
SAUSA300_1058	*hyl*	Alpha-hemolysin	−6.5	−4.0^a^
SAUSA300_2367	*hlgB*	Bi-component gamma-hemolysin HlgAB/HlgCB subunit B	−3.5	−5.7^a^
SAUSA300_1988	SAUSA300_1988	Delta-lysin family phenol-soluble modulin	−3.0	1.0
SAUSA300_0438	*aaa*	Autolysin/adhesin Aaa	−2.5	−1.7^a^
SAUSA300_2572	*aur*	Zinc metalloproteinase aureolysin	−1.9	1.2

a Denotes genes significantly differentially expressed in dual-species biofilms. All genes listed were significantly differentially expressed in mono-microbial biofilms.

Changes similar to those observed under mono-species conditions were found in dual-species biofilms (Table 3, Fig. S7). Interestingly, *S. aureus* treated with TM18 in a dual-species biofilm showed extensive dysregulation of genes, with approximately one-third of the genome significantly dysregulated (878 genes; 462 upregulated and 416 downregulated) (Table S5). Like the priming effect observed in *P. aeruginosa*, the dual-species environment primed *S. aureus* through the upregulation of various efflux pumps, including *norAB*, potentially increasing its tolerance to antimicrobial treatment. Notably, TM18 treatment caused downregulation of *S. aureus* infection-associated genes under mono- and dual-species conditions (Fig. S7).

### 2.5 TM18 exhibited anti-abscess activity in a murine skin abscess model

Testing antimicrobial agents *in vivo* is crucial for understanding their efficacy in complex biological environments. While *in vitro* studies provide initial insights into the biological activities of compounds, they cannot fully replicate the host environment, including immune system interactions with pathogens and antimicrobial agents [35,36].

Given the remarkable *in vitro* activity of lipo-peptoid TM18*,* we explored its potential in a murine skin abscess model [37,38]. Prior to skin toxicity testing (Table S10), the cytotoxicity of each peptoid was assessed using L929 mouse fibroblast cells (Table S9). The lowest observed lethal dose 50 (LC_50_) was 50 μg/mL for TM11 and TM14, while all other peptoids exhibited LC_50_ values exceeding 100 μg/mL. Additionally, an *in vivo* dose of up to 5 mg/kg (equivalent to 125 μg per injection in a 25 g mouse) showed no detectable toxicity for TM14 and TM18.

**Fig. 3:**
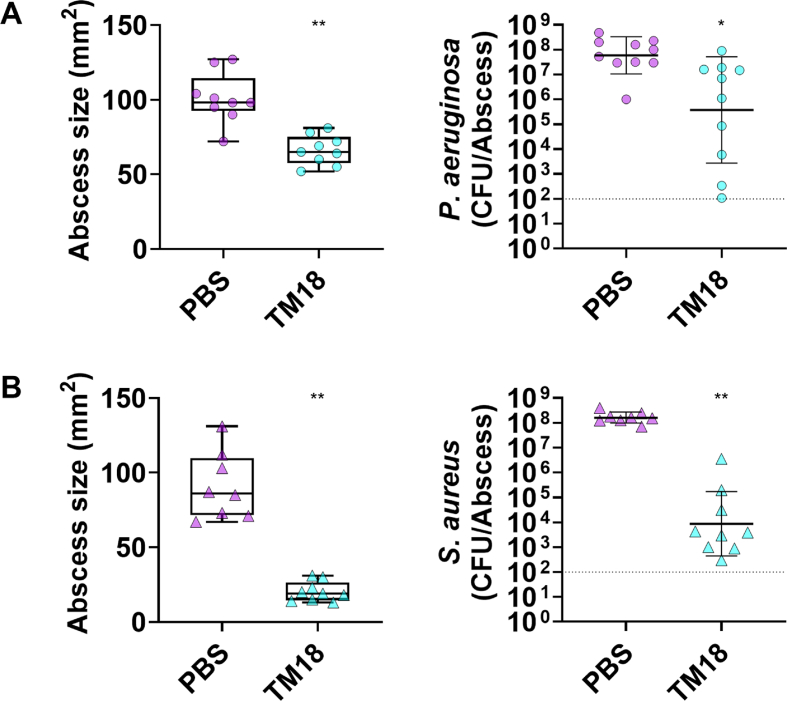
***In vivo* activity of peptoid TM18 treatment in a high-density mono-species *P. aeruginosa* or *S. aureus* infection model*.*** Female Swiss Webster mice were subcutaneously injected with 2.5 × 10^7^ CFU of (**A**) *P. aeruginosa* LESB58, or (**B**) *S. aureus* USA300 LAC. One-hour post-infection, mice were treated intra-abscess with 125 μg (5 mg/kg) peptoid or PBS as a vehicle control. After three days, the mice were euthanized. Abscesses were measured, including the area of skin dermonecrosis, and collected for bacterial enumeration. Results are presented as median with whiskers indicating min and max values, or as geometric mean ± geometric SD. Statistical significance is denoted as ∗∗*p* < 0.01, according to Kruskal−Wallis test with Dunn's correction. Dashed line represents limit of detection.

Mice were infected with either *P. aeruginosa* or *S. aureus* and treated 1 h post-infection. Peptoid TM18 significantly reduced *P. aeruginosa* skin abscesses by 35% (Fig. 3A, left) and reduced bacterial survivors by >100-fold, although this reduction was not statistically significant (Fig. 3A, right). The activity of peptoid TM18 was compared to that of the established peptoid TM1. In comparison, peptoid TM1 reduced abscesses by 30%, but showed only minor bactericidal activity. TM14 was also tested but exhibited no anti-abscess activity in this model (Fig. S8A). Additionally, TM1 and TM18 prevented scabbing at the infection site (Fig. S9A).

In *S. aureus* infections, TM18 significantly reduced abscess sizes 78% (Fig. 3B, left), prevented scabbing (Fig. S9B), and reduced bacterial survivors by more than >10,000-fold (Fig. 3B, right). TM1 showed a significant reduction of abscesses by 45%, prevented scabbing, and significantly reduced bacterial survivors by over 100-fold. TM14 did not reduce abscesses but decreased scabbing by 50% and significantly reduced *S. aureus* survivors by ∼10-fold (Figure S8B, Figure S9B).

### 2.6 TM18 and meropenem as an effective combination therapy for treating complex skin abscess co-infections by *P. aeruginosa* and *S. aureus*

Combination therapy is critically important in the treatment of infectious diseases, particularly for combating multi-drug resistant pathogens and enhancing therapeutic efficacy [39]. The challenge of finding antibiotics effective against multiple pathogens underscores the need for combination approaches, as single-agent therapies often fail to address the diverse mechanisms of resistance and virulence exhibited by various bacterial species.

To test an effective antibiotic combination with TM18 *in vivo*, we first needed to identify an antibiotic that works equally well against both bacterial pathogens to avoid eradication of one bacterium over the other. Various antibiotics from different classes against *P. aeruginosa* LESB58 and *S. aureus* USA300 LAC were tested (Table S7). We found that tobramycin (MIC 1.56 μg/mL against *P. aeruginosa*, 0.78 μg/mL against *S. aureus*) and meropenem (MIC 1.56 μg/mL against *P. aeruginosa*, 0.78 μg/mL against *S. aureus*) had similar MICs against both strains (Table S6). Both antibiotics were then tested in combination with TM18 using a checkerboard synergy assay, showing additive effects against both strains (FICI 0.75–1) (Table S7).

**Fig. 4:**
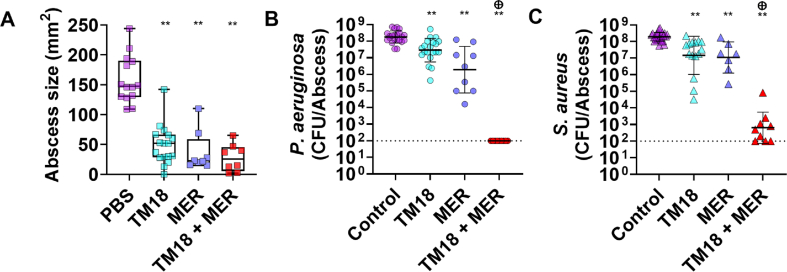
**Peptoid TM18 synergizes with meropenem against *P. aeruginosa* and *S. aureus* co-infections *in vivo.*** Female Swiss Webster mice were subcutaneously injected with 2.5 × 10^7^ CFU of *P. aeruginosa* LESB58 and *S. aureus* USA300 LAC. After 1 h, mice were treated intra-abscess with 5 mg/kg of peptoid TM18, meropenem, 5 mg/kg, a combination of peptoid TM18 and meropenem, or vehicle (PBS) control. After three days, mice were euthanized, abscesses were measured and then collected for bacterial enumeration, and (**A**) skin dermonecrosis area was measured; (**B**) *P. aeruginosa* survivors, and (**C**) *S. aureus* survivors. Results are displayed as geometric mean ± geometric SD. ∗*p* < 0.05, ∗∗*p* < 0.01, according to Kruskal−Wallis test with Dunn's correction. Sum of CFU log reduction obtained for each single treatment and a Mann-Whitney test performed to compare PAE of the individual treatments to the CFU counts obtained by the drug combination ⊕ *p* =<0.05 is synergistic. Dashed line indicates limit of detection.

Given the challenge of treating polymicrobial infections with conventional antibiotics [10,40,41], we further investigated meropenem and TM18 efficacy, individual and combined, using the *P. aeruginosa* and *S. aureus* dual-species *in vivo* infection model (Fig. 4). Individual treatments with TM18 and meropenem significantly reduced abscess sizes by 65% and 78%, respectively. The combination also reduced abscess sizes by 65% (Fig. 4A). However, in the co-infection model, TM18 lost some of its bactericidal activity against both *P. aeruginosa* and *S. aureus*, like the results obtained in the *in vitro* dual-species biofilm experiments (Fig. 1E and F), albeit still a significant reduction.

Meropenem alone significantly reduced abscess sizes by 85% and efficiently killed *P. aeruginosa* and *S. aureus* by ∼100- and 10-fold, respectively. Intriguingly, the combination of TM18 and meropenem not only significantly reduced abscess sizes but also reduced the bacterial burden of *P. aeruginosa* to the limit of detection (Fig. 4B) and reduced *S. aureus* bacterial burden to within 10-fold of the limit of detection (Fig. 4C). This effect was synergistic, highlighting the importance of testing for *in vivo* synergistic interactions. This also shows the strong potential of using peptoids in combination therapy with broad-spectrum antibiotics to effectively manage complex infections.

## 3 Discussion

The global burden of AMR is escalating, posing a significant threat to public health [42]. Given the urgent need for new antimicrobials, the ability to repurpose existing therapies and the development of new combination therapies to enhance efficacy and reduce resistance has become a promising approach to tackling antibiotic-resistant pathogens. Peptoids, a class of peptides resistant to proteases with increased biostability, potent antimicrobial activity, and low toxicity to human cells, have emerged as promising candidates for novel antimicrobial therapeutics [17,43].

**Does the addition of a cationic *N*Lys residue improve the antimicrobial activity of peptoids?** The addition of *N*Lys introduces a positive charge to the peptoid molecule, facilitating electrostatic interactions with the negatively charged bacterial membranes and promoting membrane disruption and subsequent bacterial lysis [44,45]. We hypothesized that the incorporation of an additional *N*Lys molecule would enhance the bactericidal efficacy of peptoids and improve biofilm penetration. Using an iterative process to refine previously characterized antimicrobial peptoids (TM1-TM10), we found that the addition of a cationic *N*Lys residue did not uniformly enhance the ability to combat priority pathogens. Our broad screen of ten peptoids with additional *N*Lys residues revealed that the positive charge predominantly reduced efficacy against Gram-negative *P. aeruginosa* (Table 1) compared to previously published TM1-TM10 [17]. Three out of 10 peptoids exhibited a weak two-fold decrease in activity against *P. aeruginosa*, while the remaining peptoids displayed a two-fold increase. The activity against *S. aureus* remained largely unchanged, except for TM19, which showed a four-fold increase in efficacy. Hence, host-mimicking conditions in evaluating the true potential of antimicrobial agents are crucial, as they reveal nuances in efficacy that are not apparent in standard laboratory media [46].

Peptidomimetics also hold significant potential for development as an anti-biofilm drug compound [17,23,47]. To further explore the efficacy of our modified peptoids against biofilms formed by either bacterium alone or in co-culture, we conducted biofilm eradication experiments similar to those previously performed for TM1-TM10. Intriguingly, the addition of *N*Lys enhanced anti-biofilm activity against *P. aeruginosa* while maintaining efficacy against *S. aureus* mono-species biofilms (Fig. 1). Most of the modified peptoids also retained their activity against dual-species biofilms formed by both pathogens. Notably, TM14 and TM18 demonstrated superior activity, effectively eradicating both bacteria within a dual-species biofilm. TM14 is particularly interesting because its parent compound, TM4, which features halogen substituents, did not exhibit anti-biofilm activity against *P. aeruginosa* in mono- or dual-species conditions. Similarly, lipo-peptoid TM18, an *N*Lys-modified version of TM8, a lead candidate from our previous study [17], showed improved anti-biofilm activity against both mono- and dual-species biofilms of either pathogen. Remarkably, while the potential of *N*Lys-modified peptoids was shown in previous research [45], our study is among the first to demonstrate such significant anti-biofilm efficacy in dual-species settings, underscoring the unique promise of these modifications in combating complex biofilm-associated infections.

We and others [48,49] highlight the significance of specific chemical modifications in enhancing the antimicrobial properties of peptoids, while also emphasizing the complexity of the chemical landscape and the influence of culture conditions on peptoid activity. The observed improvement in anti-biofilm activity against *P. aeruginosa* and the sustained efficacy against *S. aureus* suggest that *N*Lys-modified peptoids could be a promising avenue for developing effective treatments against polymicrobial biofilms. This emphasizes the necessity for tailored approaches in peptoid design rather than generalizing modifications, also considering the propensity for self-assembly of peptoids with alkyl tails. We have previously shown that relatively small modifications in the chemical structure of lipo-peptoids can have dramatic effects on the self-assembly [17], including the critical aggregation concentration, aggregation number and morphology of self-assembled particles. However, in this case we observe that addition of one *N*Lys group in TM18 has limited effect on the self-assembled particles when comparing to TM8.

**Optimized peptoids as alternatives to other peptide-based antibiotics**. The efficacy of TM18 *in vitro* and *in vivo* is comparable to clinically used peptide-based antibiotics like colistin and daptomycin. The MIC values of TM18 in both DFG and MHB are within two-fold range of colistin's activity against *P. aeruginosa*, while demonstrating strong activity against *S. aureus*, where colistin has no canonical activity [50]. Similarly, TM18's MIC is comparable to that of daptomycin in DFG, with the added advantage that TM18 retains activity against *P. aeruginosa*, unlike daptomycin [50]. These findings suggest that peptoids represent a promising class of broad-spectrum antimicrobials with potential advantages in treating complex polymicrobial infections.

**The co-existence of *P. aeruginosa* and *S. aureus* primes both bacteria to become more tolerant towards antimicrobial compounds.** Previous studies have shown that the presence of multiple bacterial species alters gene expression, leading to increased antibiotic tolerance [6]. Tognon et al. [51] demonstrated that co-infections under planktonic conditions result in significant metabolic changes in both organisms, specifically in nitrogen, amino acid and nucleotide metabolism. Briaud et al. [52] extended this by using RNA-Seq to show that *S. aureus* dysregulates 217 genes in the presence of *P. aeruginosa*, including genes involved in major metabolic pathways and energetic metabolism including dehydrogenases. These changes occur despite no differences in growth, suggesting a priming effect. Our biofilm studies corroborate these findings, showing similar dysregulation patterns, including dysregulation of metabolic pathways and efflux pumps (Table 2, Table 3), leading to antibiotic tolerance. In dual-species biofilms, *S. aureus* exhibits upregulation of efflux pumps, such as NorAB, which may protect it from *P. aeruginosa*-produced phenanzines that would otherwise inhibit its growth [53].

Furthermore, *P. aeruginosa* benefits from iron potentially released by *S. aureus*, thereby enhancing its survival in co-culture conditions [54]. This further supports the potential priming effect, which prepares bacteria to withstand future harsh conditions. Magalhaes et al. [55] found that *P. aeruginosa* in dual-species biofilms drives *S. aureus* into fermentation [56] and downregulates metabolic and peptidoglycan synthesis genes while upregulating membrane activity and carbon transport. Gene expression changes in *P. aeruginosa* were minimal, primarily affecting dehydrogenase activity. Miller et al. [57] observed that *P. aeruginosa* modulates only 0.3% of its genome in mixed biofilms, whereas *S. aureus* adjusts about 5%, leading to increased virulence and metabolic quiescence.

Our RNA-Seq analysis of bacterial biofilms grown under host-mimicking conditions revealed significant upregulation of the aminoarabinose biosynthesis pathway. This modification of the lipopolysaccharide (LPS) layer enhances resistance to cationic antimicrobial peptides and contributes to survival in hostile environments [58]. Additionally, there was a notable upregulation of efflux pump systems such as MexAB-OprM and MexXY-OprM, as well as porin proteins such as OprD that regulate membrane permeability. These changes suggest that *P. aeruginosa* is better equipped to survive in the presence of *S. aureus* by preemptively activating defense mechanisms that confer a higher level of tolerance and adaptability. Similarly, *S. aureus* upregulated several efflux pumps under dual-species conditions. Moreover, a widespread upregulation of oxidative stress response genes was observed in dual-species biofilms of both organisms compared to their respective mono-species biofilms. Increased ROS levels can lead to the modulation of resistance gene expression, increased mutation rates and enhanced horizontal gene transfer [59]. This further supports a priming mechanism and indicates enhanced tolerance to both host immune responses and antibiotic treatments.

**Mechanisms of TM18 in the treatment of *P. aeruginosa-S. aureus* dual-species biofilms**. We further investigated the mechanisms of TM18 at the molecular level to understand its efficacy against *P. aeruginosa* and *S. aureus* dual-species biofilms. We observed variable responses among peptoids in their ability to kill bacteria within biofilms under dual-species conditions (Fig. 1). Our RNA-seq data indicate that TM18 induces transcriptomic responses similar to those observed with conventional AMPs, demonstrating its potential as a novel AMP alternative. The similarities in bacterial responses do not necessarily indicate an identical mechanism of action, as AMP mechanisms remain diverse and not well-defined. Instead, for example, the upregulation of *pmr* and *anr* pathways aligns with general bacterial adaptations to cationic compounds rather than a specific AMP-associated mode of action.

Focusing on TM18 due to its superior activity against *P. aeruginosa* mono-species biofilms, we investigated its effects under both mono- and dual-species conditions. Interestingly, TM18 minimally impacted *P. aeruginosa* gene expression while significantly altering *S. aureus* gene expression. This aligns with our hypothesis that bacteria are primed under dual-species conditions, making TM18 more effective in mono-species settings but less so in dual-species ones. We observed dysregulation in efflux pumps, LPS and quorum sensing in *P. aeruginosa*, though expression levels were lower under dual-species conditions compared to mono-species ones. This suggests that *P. aeruginosa* biofilms, being three days old, were already more resistant/tolerant. Notably, TM18 treatment upregulated phenazine biosynthesis (Fig. S4), which is associated with increased antibiotic tolerance in *P. aeruginosa* biofilms [60]. However, this comes with a caveat: while phenazine production enhances attacks against *S. aureus*, it also inadvertently increases tolerance by upregulating efflux pumps. This dual effect complicates the overall impact of treating dual-species infections.

In contrast, gene expression analysis of *S. aureus* revealed extensive dysregulation in response to TM18 treatment, likely due to the high concentration used. We found significant dysregulation in proteases and peptidases, including an upregulation of HslV and ClpP, which are involved in stress response and protein degradation [61,62]. The downregulation of serine (Spl) proteases, which are associated with virulence and biofilm maintenance, suggests that TM18 disrupts *S. aureus* biofilm formation by targeting the expression of the *spl* operon, which warrants further investigations in the future. Additionally, there was a strong upregulation of other proteases, indicating that *S. aureus* senses the presence of the peptoid [63] but cannot degrade it due to its protease-resistant backbone structure. Other dysregulated pathways included phosphate metabolism, type VII secretion system as well as various lysins, indicating reduced virulence of *S. aureus* in the presence of TM18.

**Enhanced treatment of complex biofilm-associated infections with TM18 and antibiotic combinations.** Polymicrobial interactions are inherently complex [6] and are further complicated by the host immune system, which can reduce the efficacy of conventional antibiotics [25,64]. We explored the efficacy of TM18 in treating such infections, motivated by the promising mechanistic insights observed under dual-species biofilm conditions. TM18 demonstrated strong anti-abscess activity against mono-species *P. aeruginosa* and *S. aureus* infections, which was notable since TM8, lacking an additional *N*Lys residue, was inactive against *P. aeruginosa* in this murine model [17]. Encouraged by these results, we tested TM18 in a dual-species infection model, where its activity was reduced, potentially due to the priming effect of the co-existence of these two bacteria. Nonetheless, TM18 showed similar activity to the carbapenem antibiotic meropenem used in this co-infection model.

Given these findings, we hypothesized that combining TM18 with an antibiotic might enhance its efficacy as previously shown for other peptides [37]. We identified meropenem, used to treat infections caused by *P. aeruginosa*, as a potential candidate. Meropenem is generally not the first choice for treating *S. aureus* infections, but it might be used in combination with other antibiotics in specific cases where mixed infections are present or when other options are limited. Meropenem is a broad-spectrum carbapenem antibiotic; a common resistance mechanism in *P. aeruginosa* involves the downregulation of the porin protein OprD, which facilitates meropenem entry into the cell [65]. In mono-species biofilms, TM18 treatment resulted in a downregulation of *oprD*, indicating that meropenem might not be effective in combinatorial treatment (consistent with our synergy experiments where we only found additive activity; Table S7). However, in dual-species biofilms, *oprD* downregulation was not observed, suggesting that the dual-species environment limits the ability of *P. aeruginosa* to modulate *oprD* expression. This led us to hypothesize a potential synergistic effect between TM18 and meropenem in dual-species environments, suggesting that the dual-species environment restricts the ability of *P. aeruginosa* to modulate *oprD* expression.

Remarkably, *in vivo* studies showed that dual-species infections were significantly reduced with the combination of TM18 and meropenem, demonstrating a strong synergistic effect against both *P. aeruginosa* and *S. aureus* (Fig. 4). Previous reports have shown that antimicrobial peptides can synergize with meropenem [66] and other antibiotics [67,68], highlighting the potential of combining peptidomimetics with conventional antibiotics to enhance efficacy. Our study is among the first to show near-complete eradication of high-density infections upon such treatment.

Our study presents promising results for the broad-spectrum activity of TM18, but there are several limitations to consider. While RNA-Seq analysis provided valuable insights into the gene expression changes induced by TM18, further studies are necessary to confirm these findings and fully elucidate the molecular mechanisms involved. Additionally, the potential for resistance development against peptides, peptoids, and lipo-peptoids has not been thoroughly investigated. Long-term studies assessing the risk of resistance emergence are crucial for evaluating the sustainability of peptoids as a treatment option. To translate our findings into clinical applications, large-scale clinical trials are required to evaluate the efficacy of TM18 in treating various types of infections, including those in different anatomical sites and patient populations. Moreover, developing effective delivery systems to target infection sites will be critical for maximizing its therapeutic benefits.

In conclusion, our study highlights the potential of *N*Lys-modified peptoids as effective treatments against dual-species biofilms and antibiotic-resistant pathogens. TM18 showed promising activity against both *P. aeruginosa* and *S. aureus*. The addition of an *N*Lys residue enhanced the antibiofilm properties of TM18, and the combination of TM18 with meropenem offers a potential strategy for treating complex biofilm-associated infections. Further studies are warranted to optimize the design of *N*Lys-modified peptoids and to explore their mechanisms of action in more detail.

## 4 Materials and methods

### 4.1 Bacterial strains and growth conditions

Bacterial strains used in this study include *P. aeruginosa* LESB58 [69], PAO1 [70](Hancock lab; University of British Columbia), PA14 [71], and *S. aureus* USA300 LAC [72], Newman [73], and HG001 [74]. Strains were cultured in Lysogeny Broth (LB) or double Yeast Tryptone (dYT) for 16–24 h at 37 °C. Liquid cultures were grown with shaking at 250 rpm and growth was measured using a spectrophotometer (Eppendorf) at an absorbance OD_600_ (optical density 600 nm). Co-cultures were separated on selective agar plates: *Pseudomonas* cetrimide agar (Oxoid) to select for *P. aeruginosa* and 7.5 % NaCl plates to select for *S. aureus* as previously described [17].

### 4.2 Antimicrobial activity assay

Minimum inhibitory concentrations (MIC) were determined using an adapted broth microdilution method [75] in Greiner bio flat chimney polypropylene 96-well plates. *P. aeruginosa* was used at 1 × 10^6^ CFU/mL and *S. aureus* at 5 × 10^6^ CFU/mL in MHB (Oxoid) and DFG, respectively. A separate polypropylene plate was used to prepare two-fold peptoid dilutions in PBS pH 7.5 (Gibco). Bacterial growth was determined by visible turbidity after incubation at 37 °C for 16 h (*S. aureus*) or 24 h (*P. aeruginosa*). MIC values were determined as the lowest concentration of the drug where no bacterial growth was visible. Metabolic activity (color change from blue to pink) was further determined by adding the metabolic dye PrestoBlue™ (Invitrogen) to each well for 30 min as confirmation of visual determination. All tests were performed in triplicate.

### 4.3 Small angle X-ray scattering (SAXS)

SAXS experiments on peptoid TM18 was performed at ALS beamline 12.3.1 LBNL Berkeley, California, USA [76], with a detector distance of 2 m and X-ray wavelength of λ = 1.27 Å, covering a Q range of 0.009 Å^−1^ to 0.4 Å^−1^. The data set was calibrated to an absolute intensity scale using water as a primary standard. All experiments were performed at room temperature and data were processed as previously described [77]. The scattering data for TM18 was analyzed using a core-shell ellipsoidal model previously described in detail by Jensen et al. [78].

### 4.4 Biofilm growth conditions

Greiner bio flat chimney polypropylene 96-well plates were used to grow biofilms in host-mimicking media, consisting of Gibco Dulbecco's minimal Eagle's medium (DMEM; Thermo Fisher) supplemented with 5 % fetal bovine serum (FBS; NZ origin) and 1 % glucose (DFG). Briefly, bacteria were scraped from an overnight-grown plate, resuspended in PBS (Gibco, pH 7.4), and adjusted to a final CFU/mL in DFG broth: *P. aeruginosa* 1 × 10^7^ CFU/mL and *S. aureus* 1 × 10^7^ CFU/mL. For dual-species biofilms of *P. aeruginosa* and *S. aureus* a 1:1 ratio was used with 1 x 10^7^ CFU/mL per species.

### 4.5 Biofilm eradication activity of peptoids

The biofilm eradication methodology was adapted from Nielsen et al. [17]. Mono-species and dual-species biofilms were grown for 20–24 h in DFG in 96-well microtiter plates as above. Biofilms were washed with PBS once and fresh DFG added to the wells. A separate polypropylene plate was used to prepare the two-fold peptoid dilutions. Subsequently, peptoids (dilutions or a 31.25 μg/mL fixed concentration) were added to the biofilm plate. A fixed concentration of 31.25 μg/mL was used as an antibiofilm concentration as it effectively reduced polymicrobial biofilms across all tested peptoids (Fig. S1) and aligns with our previous work on structurally related parent peptoids [17]. The treated plate was incubated for another 20–24 h at 37 °C. After incubation, plates were washed three times with PBS and stained with 0.1 % crystal violet (CV) (Sigma Aldrich) for 20–30 min with shaking at 150 RPM. Plates were washed with PBS three to five times to remove unbound CV. The remaining dye (i.e., biofilm mass) was dissolved in 70% EtOH for 20–30 min shaking at 150 RPM. The OD_595_ was measured with a plate reader (Thermo Scientific Varioskan® Flash).

For eradication experiments at a fixed concentration, biofilms were scraped from five technical replicate wells with a cotton swab, submerged in one mL of PBS, vortexed and used for serial dilutions. Dilutions were plated onto LB agar for mono-species biofilm survivors and selective agar plates for dual-species biofilms. After 24 h incubations, colonies were enumerated and presented as CFU/mL. Experiments were reproduced with at least three biological replicates.

### 4.6 Transcriptome sequencing (RNA-seq) of biofilms treated with peptoid TM18

To determine their transcriptomic responses to peptoid treatment, individual *P. aeruginosa* LESB58 or *S. aureus* USA300 LAC biofilms as well as dual-species biofilms using *P. aeruginosa* LESB58 and *S. aureus* USA300 (seeded at 1 × 10^7^ CFU/mL) were grown on four hydroxyapatite (HA) [79] discs (0.5′ diameter x 0.04–0.15′ thick) (Clarkson Chromatography Products Inc.) submerged in DFG broth in a 6-well plate for 72 h. Medium was exchanged daily. Subsequently, biofilms were treated with either PBS or 32 μg/mL TM18 for 1 h. After treatment, three HA discs were submerged in RNAprotect® (QIAGEN) to extract RNA. HA discs were soaked in a lysostaphin 12.5 μg/mL (Biovendor), and lysozyme 500 μg/mL (Sigma Aldrich) TE buffer pH 8.0 (Invitrogen) solution during sonication for 5 min at 100% intensity (40 kHz, BactoSonic waterbath sonicator) and subsequently incubated at 37 °C for 1 h. RNA was isolated using the QIAGEN RNeasy kit, according to the manufacturer's protocol. A 1-h timepoint was chosen to show the early adaptive responses, as longer exposure increased bacterial killing (Fig. S10), especially for *S. aureus. P. aeruginosa* LESB58 and *S. aureus* USA300 were selected due to their co-existence in HA disc biofilms [50], while dual-species biofilms with *P. aeruginosa* PA14 or PAO1 led to rapid S. aureus killing (Fig. S11).

Four biological replicates were used to prepare RNAseq TruSeq stranded RNA libraries from rRNA-depleted RNA samples using a Zymo-Seq RiboFree total RNA library kit and sequenced as 100 bp single-end reads (average of 11 million reads per sample, Table S1) using an Illumina NextSeq2000 at the Otago Genomics Facility (Dunedin, New Zealand). Reads were trimmed using Trimmomatic v0.39 [80] and subsequently mapped using STAR v2.7.8a [81] to either *Pseudomonas aeruginosa* LESB58 (genome assembly accession ASM2664v1, GenBank assembly GCA_000026645.1 [82]) or *Staphylococcus aureus* USA300 FPR3757 (genome assembly accession ASM1346v1, GenBank assembly GCA_000013465.1 [83]). MultiQC v1.11 [84] was used to examine mapping statistics and read quality. Differential gene expression analyses were carried out with R version 4.3.1 [85], using DESeq2 v1.40.2 [86]. Significantly differentially expressed genes were only considered from genes with >1.5-fold change and a false discovery rate (FDR) adjusted *p*-value ≤0.05. Gene ontology (GO) enrichment analysis was carried out using clusterProfiler v4.8.3 [87], *P. aeruginosa* LESB58 gene ontology terms were obtained from pseudomonas.com [88], *S. aureus* USA300 LAC gene ontology terms were obtained from UniProtKB for taxonomic groups (taxonomy_id:367830) [89]. Significantly enriched terms from differentially expressed genes were plotted using gene ratio of the number of genes associated with the given ontology term, all plots were generated using ggplot2 v3.3.5 [90].

### 4.7 Checkerboard titration assays (*in vitro* synergy)

The synergy between TM18 and the antibiotics meropenem, tobramycin, ciprofloxacin, gentamicin, azithromycin, and ceftazidime (Sigma Aldrich) was determined using the checkerboard titration assay [91]. Each compound was serially diluted in PBS before transferring to a 96-well plate containing 1 × 10^6^ CFU/mL *P. aeruginosa* or *S. aureus*, respectively, in DFG medium and incubated at 37 °C for 16–24 h. The MIC or combinatorial effect was determined as the lowest concentration with no visible growth and the fractional inhibitory concentration index (FICI) was calculated as [(MIC_A_ in combination)/MIC_A_] + [(MIC_B_ in combination)/MIC_B_] as previously [92]. FICIs ≤ 0.5 indicate synergy, 0.5–1 indicate additivity and values above 1 indicate indifference or antagonism.

### 4.8 Peptoid toxicity assay

Peptoid toxicity was determined using the Promega CytoTox® Non-Radioactive Cytotoxicity Assay Kit. Mouse fibroblast cells (L929 cells) were seeded into 96-well plates (Greiner Bio Cell) at 10,000 cells/well. Cells were grown for 24 h in Dulbecco's minimal Eagle's medium (DMEM) high glucose + 10% fetal bovine serum (Gibco) and 1% pen-strep (Gibco) at 37 °C with 5% CO_2_. After 24 h media was removed, and cells were treated with peptoids dissolved in PBS. LDH control wells and PBS control wells were used as 100% and 0% death controls, respectively. Lethal Concentration 50 (LC_50_) was determined as the lowest concentration of peptoid which induced a minimum of 50% cell death.

### 4.9 Study approval and animals

All animal experiments were conducted in adherence to the University of Otago Animal Ethics Committee under protocol number AUP19-125. For this study, female Swiss Webster mice, aged approximately 6–8 weeks were used. These mice were obtained from the University of Otago Biomedical Research Facility. They were housed in groups of five littermates.

### 4.10 Skin abscess model and treatment with peptoids

The abscess model was performed as described previously [37,38] for mono-species infections and modified as follows for dual-species infection. *P. aeruginosa* and *S. aureus* were grown individually to an OD_600_ of 1.0 in dYT broth. Bacterial cells were washed twice in PBS pH 7.4 (Gibco) and resuspended to an OD_600_ of 2.0 for *S. aureus* (∼2.5 × 10^7^) and 1.0 for *P. aeruginosa* (∼2.5 × 10^7^). For co-infections, *P. aeruginosa* and *S. aureus* were mixed (1:1) immediately before injecting 50 μL subcutaneously into the right side of the dorsum. TM1, TM14, TM18, or sterile PBS were injected directly into the infected area 1-h post-infection at a concentration of 5 mg/kg which was the highest concentration determined as non-toxic in our murine model (Table S10). The progression of the infection was monitored daily. A caliper was used to measure the abscess lesion size manually. The SilhouetteStar™ 3D wound camera and SilhouetteConnect™ software were used to photograph, measure, and analyze abscess lesion sizes (visible dermonecrosis). The skin abscesses (including accumulated pus) were excised and homogenized in sterile PBS using SPEX®SamplePrep 1600 MiniG™ for 5 min. Serial dilutions were plated on LB agar plates for mono-species infections and selective agar plates for co-infections. Bacterial numbers were enumerated after incubation for 16–24 h at 37 °C.

### 4.11 Statistical analysis

*In vitro* experiments were performed with at least three biological replicates. *In vivo* experiments were conducted with three biological replicates with at least three mice per group. Statistical analysis was performed using GraphPad Prism v9.5.0 (GraphPad Software, Boston, Massachusetts USA). A One-way ANOVA Kruskal-Wallis test with Dunn's correction was used for mono-species biofilms. A two-way ANOVA with post-hoc Dunnett's multiple correction test was utilized for dual-species biofilms. Data was considered significant when *p*-values were below 0.05 or 0.01 as indicated. The predicted additive effect (PAE) was determined by calculating the sum of CFU log reduction for each individual treatment. The Mann-Whitney test was then performed to compare the PAE with the CFU counts obtained from the drug combination. Synergy was defined as *p* ≤ 0.05 [37,93].

## CRediT authorship contribution statement

**Samuel J.T. Wardell:** Writing – review & editing, Writing – original draft, Visualization, Software, Investigation, Formal analysis. **Deborah B.Y. Yung:** Visualization, Methodology, Investigation. **Josefine E. Nielsen:** Writing – review & editing, Investigation. **Rajesh Lamichhane:** Investigation. **Kristian Sørensen:** Resources, Investigation. **Natalia Molchanova:** Resources. **Claudine Herlan:** Resources. **Jennifer S. Lin:** Writing – review & editing, Resources. **Stefan Bräse:** Supervision. **Lyn M. Wise:** Writing – review & editing, Supervision, Funding acquisition. **Annelise E. Barron:** Writing – review & editing, Supervision, Resources. **Daniel Pletzer:** Writing – review & editing, Writing – original draft, Supervision, Resources, Project administration, Methodology, Funding acquisition, Conceptualization.

## Data and materials availability

All data needed to evaluate the conclusions in the paper are present in the paper and/or the supplementary materials. Raw RNA-seq files are available under SRA accession number PRJNA1113372.

## Declaration of competing interest

The authors declare the following financial interests/personal relationships which may be considered as potential competing interests:

• Maurice Wilkins Centre for Molecular Biodiscovery MWC4064 (DP, LW)
• Royal Society of New Zealand Marsden Fund MFP-UOO2203 (DP, LW, RL)
• University of Otago Research Grant (DP)
• Lotteries Health Postdoctoral Research fellowship, LHR-2023-215235 (SJTW)
• University of Otago doctoral scholarship (DBYY)
• NIH Director's Pioneer Award, 1DP1 OD029517 (AEB, KBS, JSL)
• SENS Research Foundation, Stanford University's Discovery Innovation Fund, the Cisco University Research Program Fund, and the Silicon Valley Community Foundation, and Dr. James J. Truchard and the Truchard Foundation (AEB)
• Novo Nordisk Foundation, and the Stanford Bio-X Program, NNF21OC0068675 (JEN)

## Data Availability

Raw RNAseq fastq files are available under SRA accession PRJNA1113372. A summary of mapping statistics is shown in Table S1. Pairwise comparisons of differentially expressed genes in mono-species *P. aeruginosa* and *S. aureus* biofilms treated with TM18 are shown in Tables S2 and S3. Differentially expressed genes in dual-species biofilms treated with TM18 for either *P. aeruginosa* or *S. aureus* are provided in Tables S4 and S5.
