# Supplementary material for: A biofilm-targeting lipo-peptoid to treat Pseudomonas aeruginosa and Staphylococcus aureus co-infections
Source: Biofilm. 2025 Mar 12;9:100272. doi: 10.1016/j.bioflm.2025.100272 (PMC12005307; doi:10.1016/j.bioflm.2025.100272)
Supplement: Multimedia component 1 [file mmc1.docx]

**Table S1. Summary of RNA-seq mapping statistics to *P. aeruginosa* LESB58 and *S. aureus* USA300.**

| **Sample Name** | **% Aligned** | **Aligned reads (M)** | **Total reads (M)** |
| --- | --- | --- | --- |
| ***P. aeruginosa* LESB58** | | | |
| PA_NT_1 | 90.40% | 13.4 | 14.8 |
| PA_NT_2 | 96.10% | 10.4 | 10.9 |
| PA_NT_3 | 93.80% | 10.6 | 11.6 |
| PA_NT_4 | 85.20% | 11.4 | 13.4 |
| PA_TM18_1 | 92.90% | 9.7 | 11.3 |
| PA_TM18_2 | 96.50% | 8.6 | 8.9 |
| PA_TM18_3 | 95.10% | 9.2 | 9.6 |
| PA_TM18_4 | 93.70% | 10.2 | 10.9 |
| PaSa_NT_1 | 10.40% | 1 | 9.8 |
| PaSa_NT_2 | 6.70% | 0.7 | 11.2 |
| PaSa_NT_3 | 11.80% | 1.3 | 10.7 |
| PaSa_NT_4 | 11.00% | 1.3 | 11.8 |
| PaSa_TM18_1 | 21.90% | 2.3 | 10.6 |
| PaSa_TM18_2 | 40.40% | 4.5 | 11 |
| PaSa_TM18_3 | 31.60% | 3 | 9.5 |
| PaSa_TM18_4 | 20.40% | 1.9 | 9.2 |
| ***S. aureus* USA300** | | | |
| Sa_NT_1 | 97.90% | 12.1 | 12.3 |
| Sa_NT_2 | 97.60% | 10.6 | 10.9 |
| Sa_NT_3 | 97.50% | 10.8 | 11.1 |
| Sa_NT_4 | 97.60% | 11.2 | 11.5 |
| Sa_TM18_1 | 95.10% | 10.9 | 11.5 |
| Sa_TM18_2 | 95.10% | 10.7 | 11.2 |
| Sa_TM18_3 | 94.10% | 10.4 | 11 |
| Sa_TM18_4 | 94.00% | 11.5 | 12.3 |
| PaSa_NT_1 | 86.70% | 8.5 | 9.8 |
| PaSa_NT_2 | 90.70% | 10.2 | 11.2 |
| PaSa_NT_3 | 85.50% | 9.1 | 10.7 |
| PaSa_NT_4 | 86.60% | 10.2 | 11.8 |
| PaSa_TM18 _1 | 66.90% | 7.1 | 10.6 |
| PaSa_TM18 _2 | 53.10% | 5.9 | 11 |
| PaSa_TM18 _3 | 59.40% | 5.6 | 9.5 |
| PaSa_TM18 _4 | 72.00% | 6.6 | 9.2 |

**Table S2-S5. Differentially expressed genes provided as Supporting Information Dataset.**

**Table S2. Pairwise comparisons of differentially expressed genes in monospecies *P. aeruginosa* biofilms treated with TM18.**

**Table S3. Pairwise comparisons of differentially expressed genes in monospecies *S. aureus* biofilms treated with TM18.**

**Table S4. Differentially expressed genes in dual-species biofilms treated with TM18 for *P. aeruginosa*.**

**Table S5. Differentially expressed genes in dual-species biofilms treated with TM18 for *S. aureus*.**

**Table S6. Antimicrobial susceptibility of various antibiotics against *P. aeruginosa* LESB58 and *S. aureus* USA300 LAC.**

| MIC (μg/mL) | AZM | CAZ | CIP | GEN | MEM | TOB | TM18 |
| --- | --- | --- | --- | --- | --- | --- | --- |
| *P. aeruginosa* LESB58 | 25 | 12.50 | 3.13 | 7.81 | 1.56 | 1.56 | 3.13 |
| *S. aureus* USA300 LAC | 125 | 31.25 | 15.63 | 0.78 | 0.78 | 0.78 | 1.56 |

**Table S7. Synergy experiments with TM18 combined with meropenem or tobramycin against *P. aeruginosa* LESB58 and *S. aureus* USA300 LAC.** FICI of >0.5 and <=1 indicates additive effect.

| Antibiotic combination with TM18: | | **MEM** | **TOB** | **CIP** | **GEN** | **AZM** | **CAZ** |
| --- | --- | --- | --- | --- | --- | --- | --- |
| **FICI** | *P. aeruginosa* | 1 | 1 | 1 | 1 | 0.625 | 0.75 |
|  | *S. aureus* | 0.75 | 1 | 0.75 | 1 | 1 | 1 |
| **Fold decrease in TM18 MIC** | *P. aeruginosa* | 2 | 2 | 1 | 1 | 8 | 2 |
|  | *S. aureus* | 2 | 1 | 4 | 1 | 1 | 1 |
| **Fold decrease in antibiotic MIC** | *P. aeruginosa* | 2 | 2 | 2 | 2 | 2 | 4 |
|  | *S. aureus* | 4 | 1 | 2 | 2 | 1 | 1 |

**Table S8. MIC (µg/mL) of TM18 to other strains of *P. aeruginosa* and *S. aureus***

|  | **TM18** |
| --- | --- |
| ***P. aeruginosa* PAO1** | |
| **DFG** | 12.5 |
| **MHB** | 6.25 |
| *P.* ***aeruginosa* PA14** | |
| **DFG** | 3.13 |
| **MHB** | 6.25 |
| ***S. aureus* HG001** | |
| **DFG** | 1.6 |
| **MHB** | 3.13 |
| ***S. aureus* Newman** | |
| **DFG** | 0.8 |
| **MHB** | 1.6 |

**Table S9. LC50 of peptoids against L929 mouse fibroblast cells.** Cells were treated for 24 h. LC_50_ determined as the concentration (μg/mL) of peptoid required to kill >50% of cells relative to PBS

|  | TM 11 | TM 12 | TM 13 | TM 14 | TM 15 | TM 16 | TM 17 | TM 18 | TM 19 | TM 20 | TM 22 |
| --- | --- | --- | --- | --- | --- | --- | --- | --- | --- | --- | --- |
| LC_50_ (μg/mL) | 50 | >100 | >100 | 50 | >100 | >100 | >100 | >100 | >100 | >100 | >100 |

**Table S10. Peptoid *in vivo* murine skin toxicity (n=2).** Data from TM1, TM4, and TM8 are from Nielsen *et al* 2022 (10.1021/acsinfecdis.1c00536)

| **Peptoid** | **2.5 mg/kg** | **5 mg/kg** | **10 mg/kg** |
| --- | --- | --- | --- |
| TM1 (original peptoid) | non-toxic | necrotic | necrotic |
| TM4 (parent of TM14) | non-toxic | inflamed, necrotic | inflamed, necrotic |
| TM8 (parent of TM18) | necrotic | necrotic | Inflamed, necrotic |
| **TM14** | non-toxic | non-toxic | inflamed |
| **TM18** | non-toxic | non-toxic | inflamed |

**
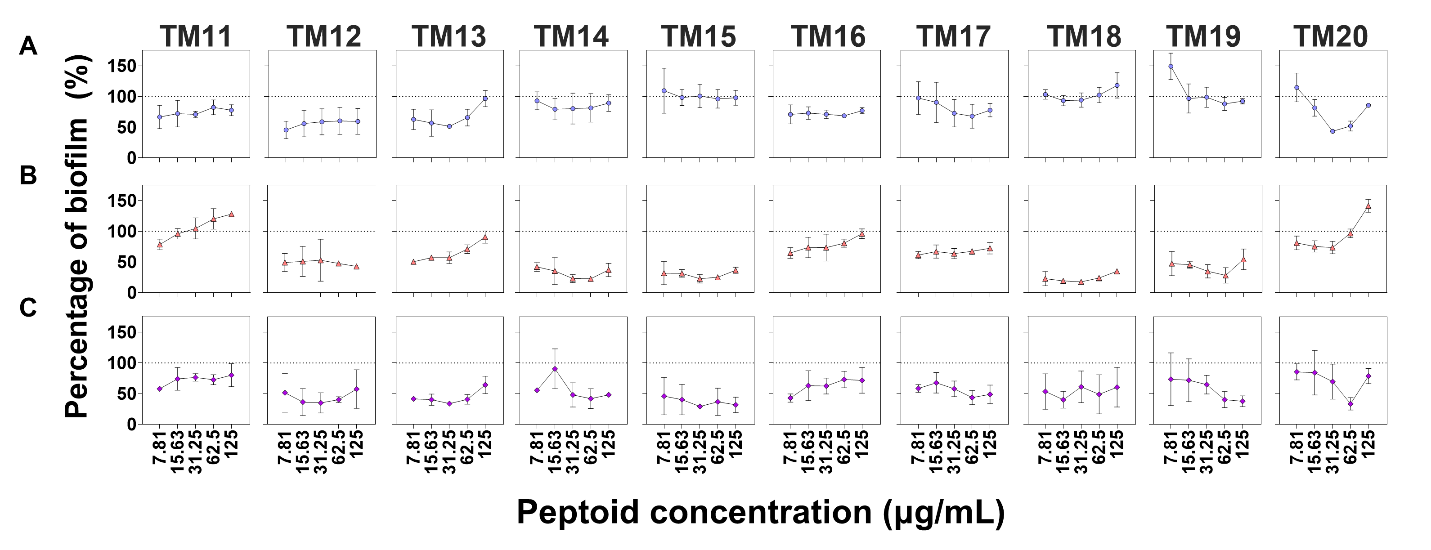
**

**Figure S1. Biofilm eradication efficacy of peptoids against monospecies (A) *P. aeruginosa* LESB58, (B) *S. aureus* USA300 LAC, and (C) dual-species biofilms *P. aeruginosa-S. aureus* in microtiter plates.** Biofilms were grown in host-mimicking media DFG for 20-24 h prior to treatment with different concentrations of peptoids TM11-TM20 (7.18-125 µg/ml) for 24 h. Biofilms were stained with 0.1% CV and normalized to the growth control, which is indicated as a dotted line in the figure. Data from three independent experiments are presented as the mean ± SEM, n=3.


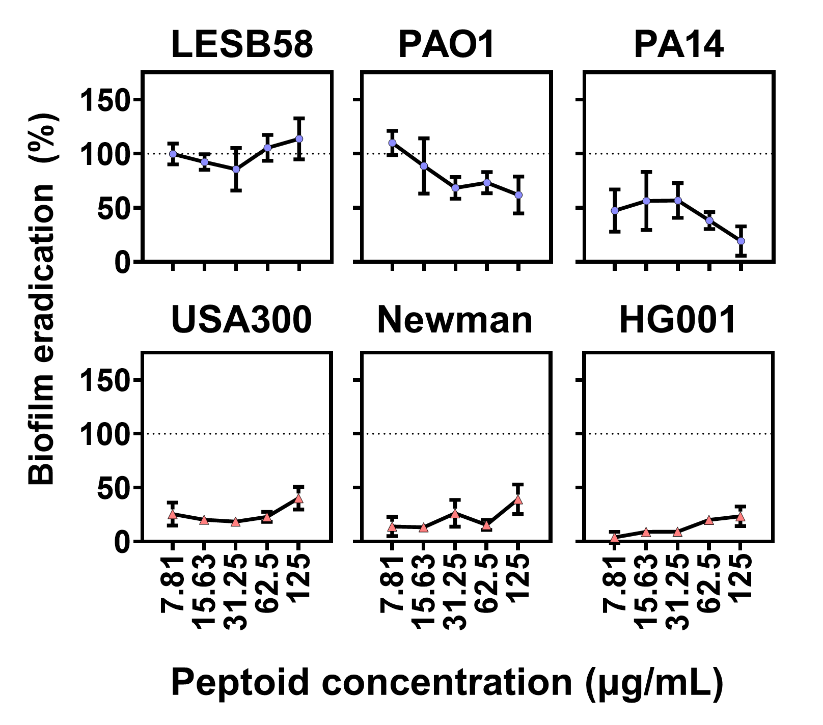


**Figure S2. Biofilm eradication efficacy of TM18 against monospecies *P. aeruginosa* or *S. aureus* biofilms.** Biofilms were grown in host-mimicking media DFG for 20-24 h prior to treatment with different concentrations of TM18 (7.18-125 µg/ml) for 24 h. Biofilms were stained with 0.1% CV and normalized to the growth control, which is indicated as a dotted line in the figure. Data from three independent experiments are presented as the mean ± SEM, n=4.


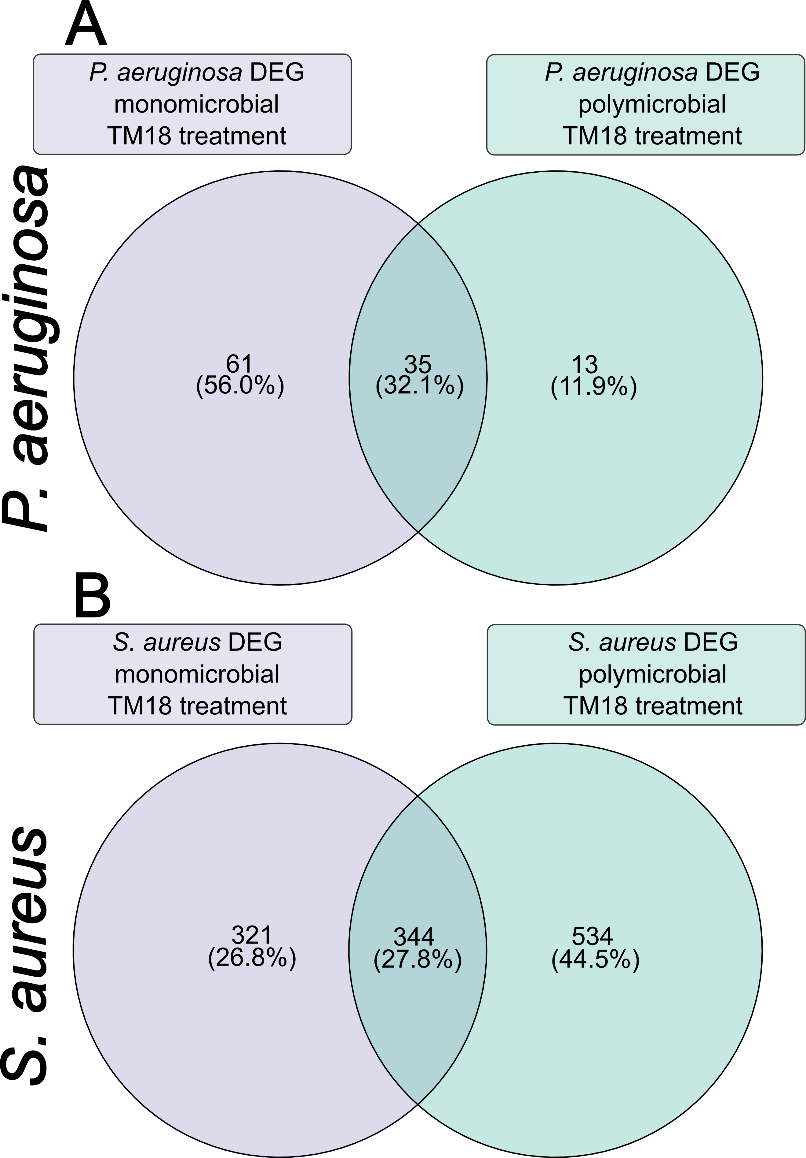


**Figure S3. Differentially expressed genes (DEG) in mono- and poly-microbial TM18 treatments show overlap.** (**A**) *P. aeruginosa* significantly differentially expressed genes under monospecies biofilms, and dual-species biofilms treated with TM18. (**B**) *S. aureus* significantly differentially expressed genes under monospecies biofilms, and dual-species biofilms treated with TM18. Significantly differentially expressed genes were identified as those with a fold-change >1.5(±), and an FDR-adjusted *p* value < 0.05, percentages (%) represent the proportion of significantly dysregulated genes.


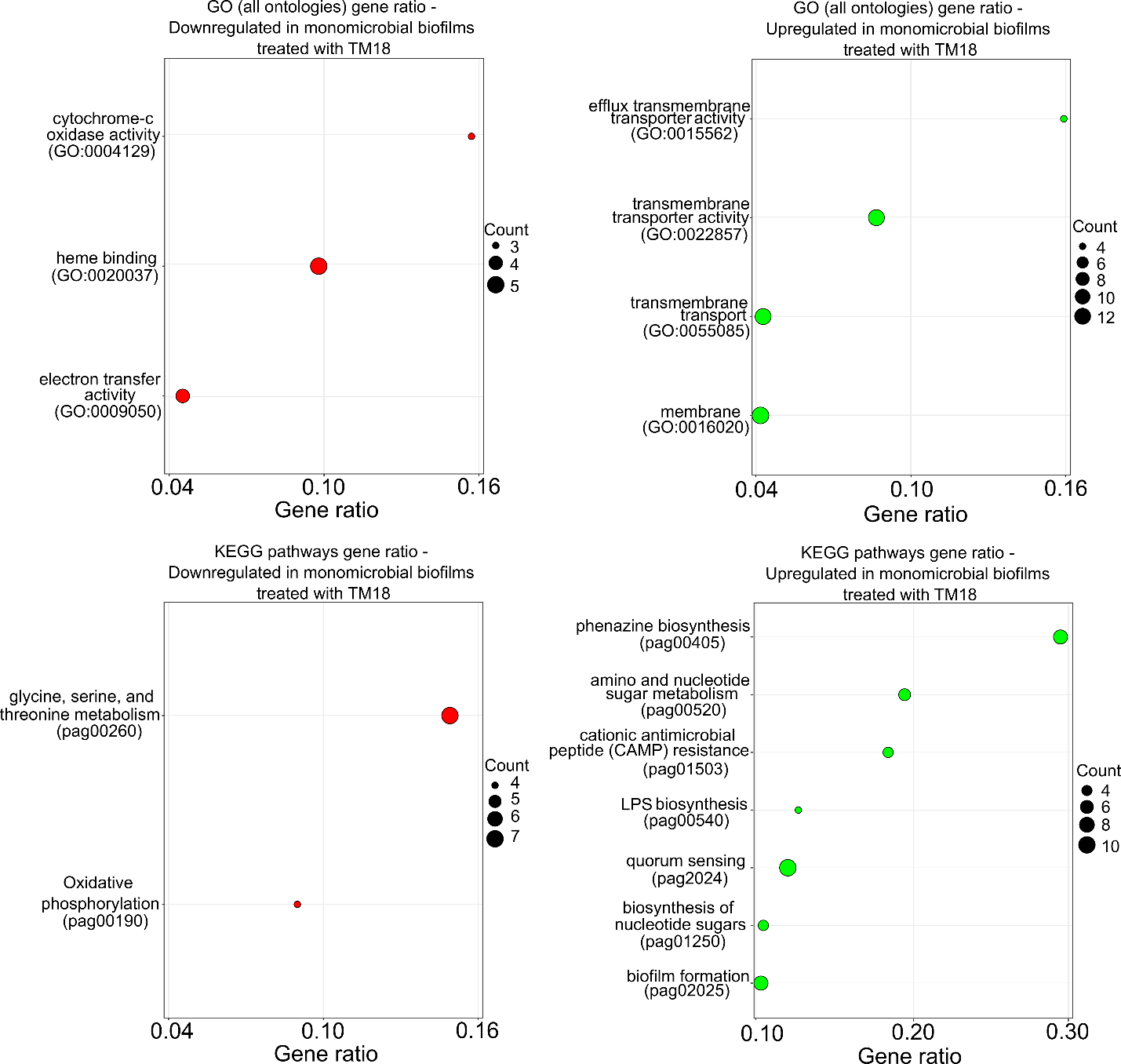


**Figure S4. Significantly enriched terms for *P. aeruginosa* monospecies biofilms treated with TM18.** Significantly enriched Gene Ontology (GO) terms, and KEGG pathways, upregulated (increased expression in TM18 treatment compared to untreated), and downregulated (decreased expression in TM18 treatment compared to untreated). Gene ratio represents proportion of genes significantly differentially expressed from the specific ontology (e.g., gene ratio of 0.5, indicates 50% of genes associated with that term are significantly dysregulated).


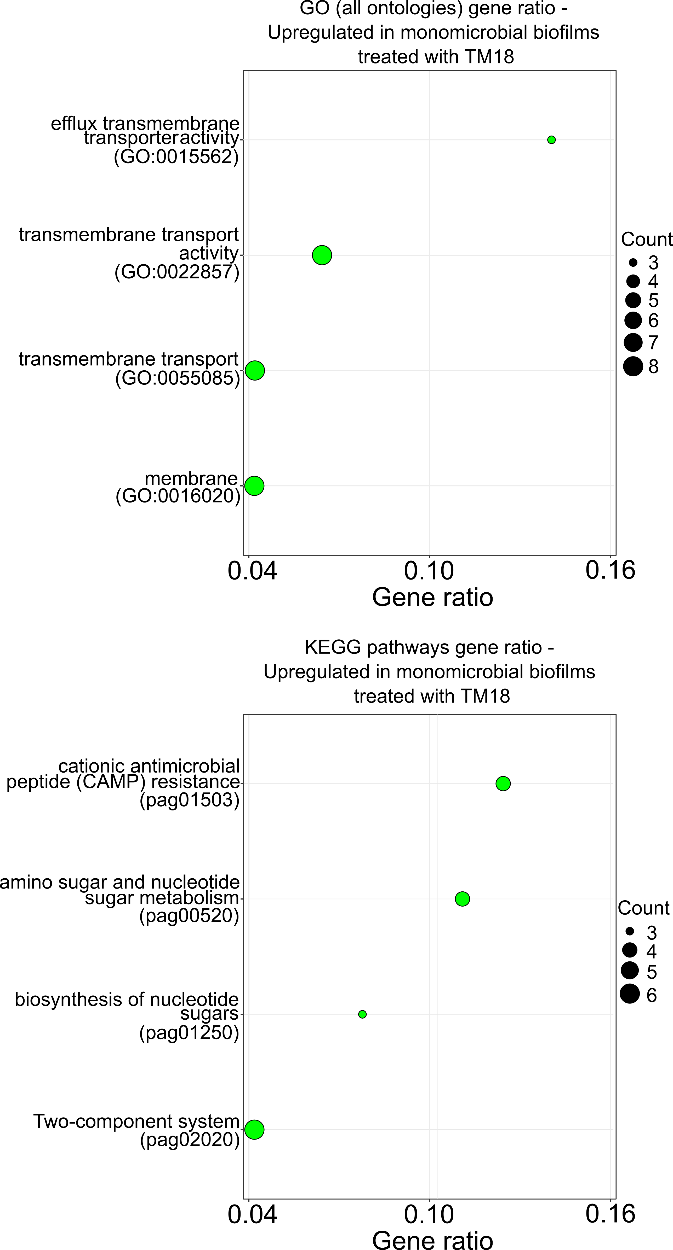


**Figure S5. Significantly enriched terms for *P. aeruginosa* dual-species biofilms treated with TM18.** Significantly enriched Gene Ontology (GO) terms, and KEGG pathways, upregulated. There were no significantly enriched terms for downregulated genes.


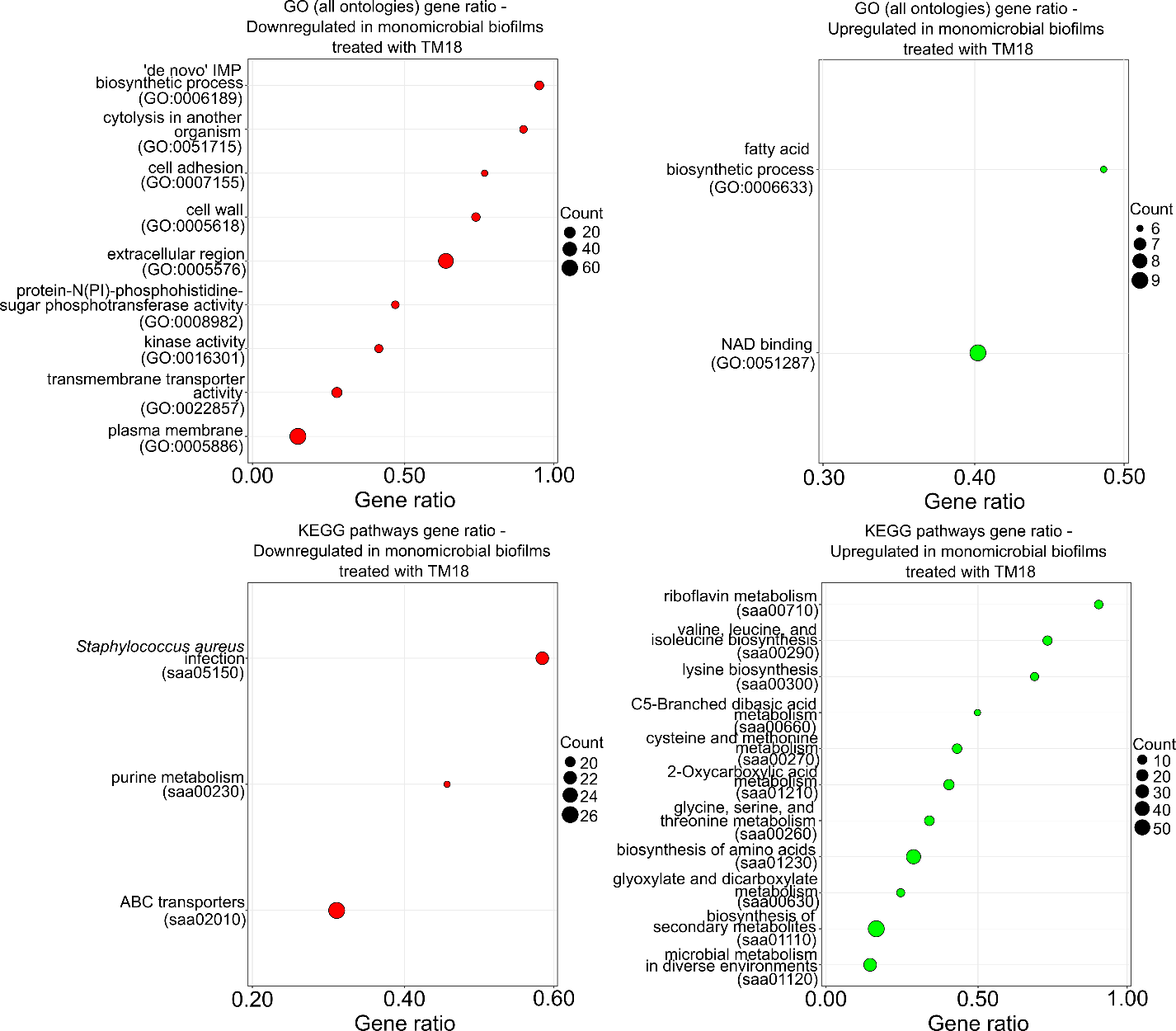


**Figure S6. Significantly enriched terms for *S. aureus* monospecies biofilms treated with TM18.** Significantly enriched GO terms, and KEGG pathways, upregulated and downregulated. Gene ratio represents proportion of genes significantly differentially expressed from the specific ontology.

**
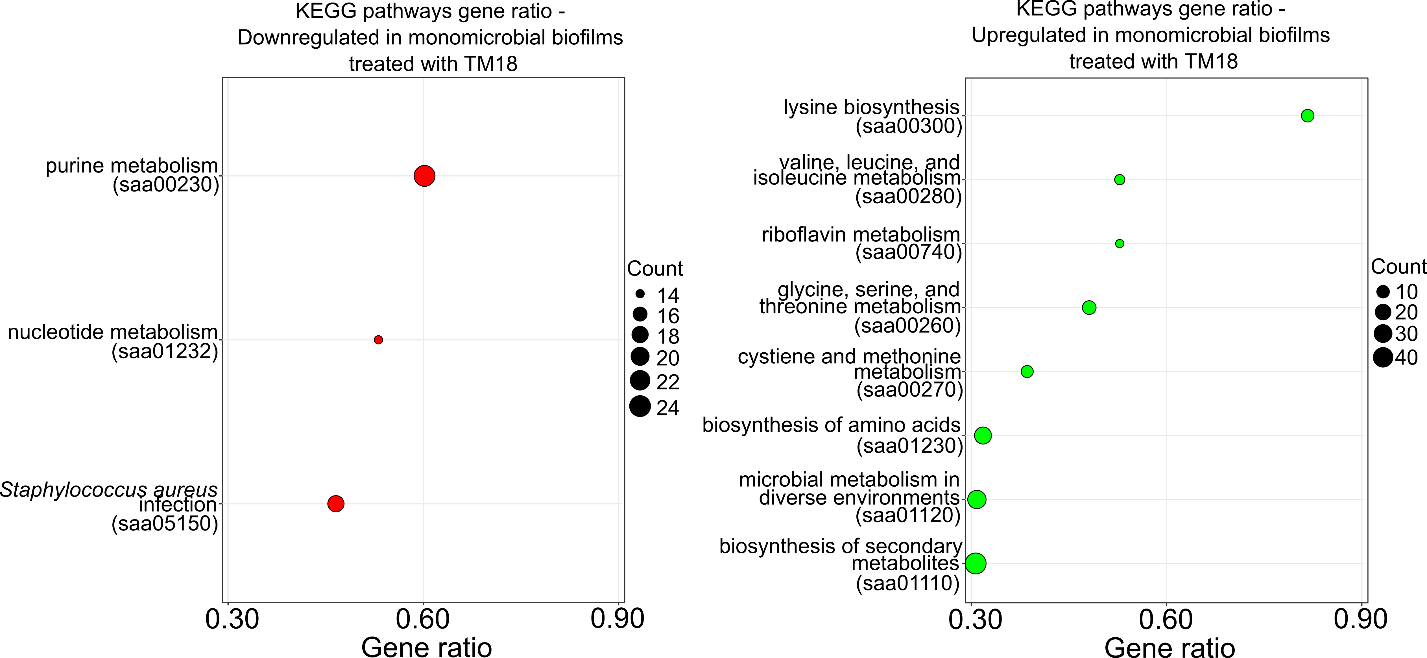
**

**Figure S7. Significantly enriched terms for *S. aureus* dual-species biofilms treated with TM18.** Significantly enriched KEGG pathways, upregulated and downregulated. There were no significantly enriched GO terms.

**
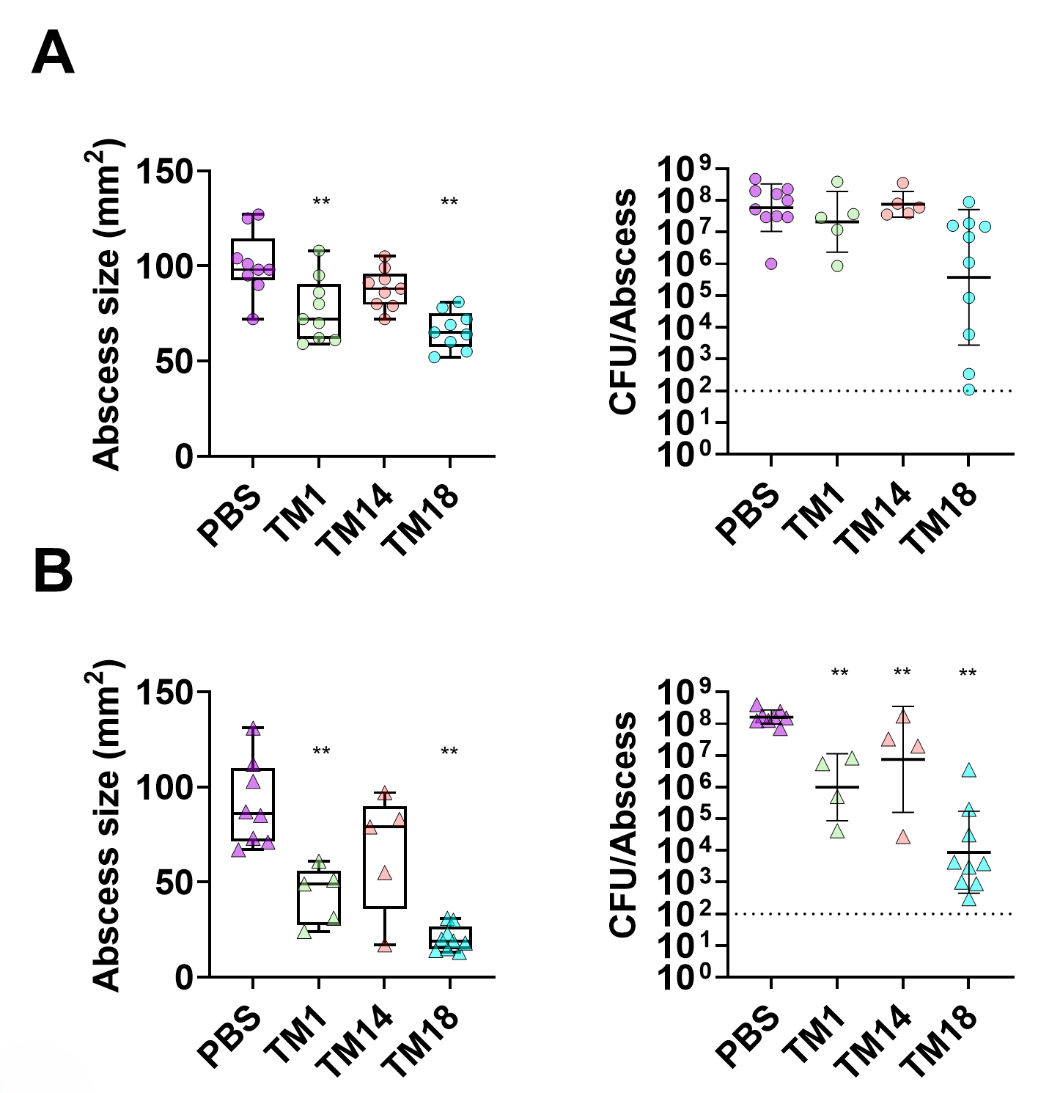
**

**Figure S8. *In vivo* activity of peptoid treatment using a high-density *P. aeruginosa* and *S. aureus* infection*.*** Female Swiss Webster mice were subcutaneously injected with 2.5 × 10^7^ CFU of (**A**) *P. aeruginosa* LESB58, or (**B**) *S. aureus* USA300 LAC. After one hour, mice were treated intra-abscess with 125 µg (5 mg/kg) peptoid or vehicle (PBS) control. After three days, mice were euthanized, abscesses were measured and then collected for bacterial enumeration, and skin dermonecrosis area measured. Results are displayed as median with whiskers to min and max or geometric mean ± geometric SD. **p* < 0.05, ***p* < 0.01, according to Kruskal−Wallis test with Dunn’s correction.

**
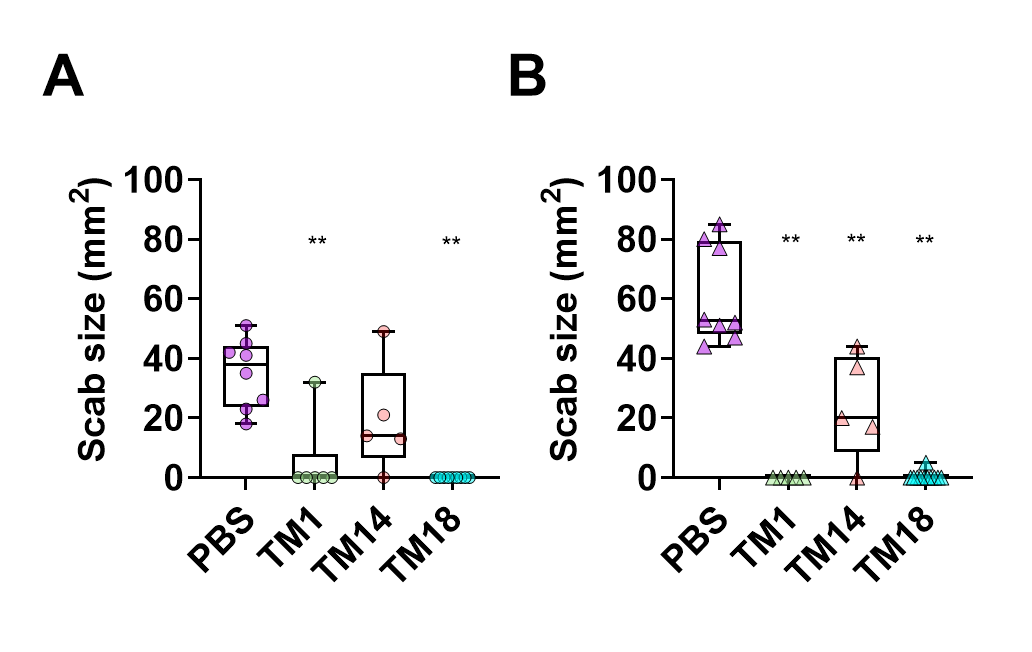
**

**Figure S9. Peptoids decrease wound scabbing.** Female Swiss Webster mice were subcutaneously injected with 2.5 × 10^7^ CFU of (**A**) *P. aeruginosa* LESB58 or (**B**) *S. aureus* USA300 LAC. After one hour, mice were treated intra-abscess with 125 µg (5 mg/kg) peptoid or vehicle (PBS) control. After three days, mice were euthanized, and scab formation was measured. Results are displayed as median with whiskers to min and max. ***p* < 0.01, ****p* < 0.001 according to Kruskal−Wallis test with Dunn’s correction.

**
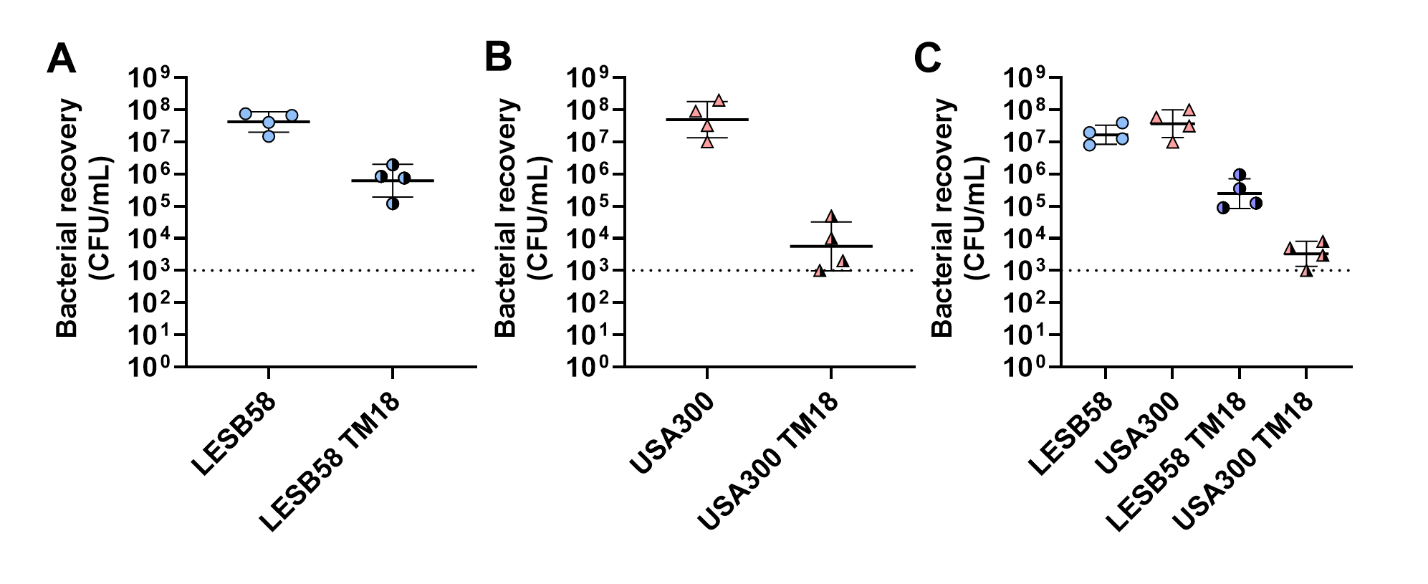
**

**Figure S10. Bacterial survival on hydroxyapatite biofilms treated with TM18 (A) monospecies *P. aeruginosa* LESB58, (B) monospecies *S. aureus* USA300 LAC, and (C) dual-species biofilms *P. aeruginosa-S. aureus*.** LESB58 and *S. aureus* USA300 (seeded at 1 × 10^7^ CFU/mL) were grown on hydroxyapatite discs submerged in DFG for 72 hours. CFUs were enumerated per disc before treatment with 32 µg/mL TM18 and after 24 h of TM18 treatment.


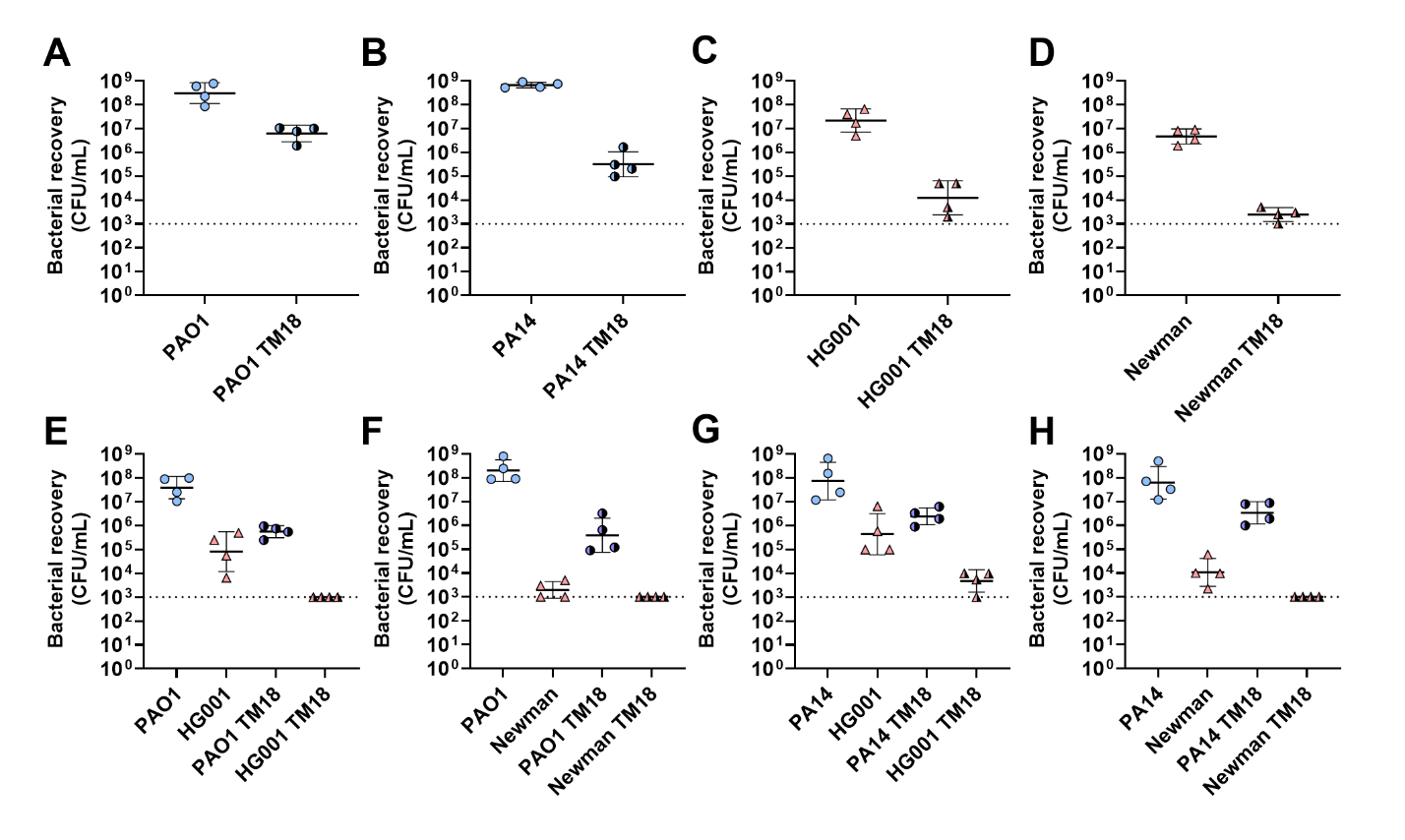


**Figure S11. Bacterial survival on hydroxyapatite biofilms treated with TM18 (A) monospecies *P. aeruginosa* PAO1, (B) monospecies *P. aeruginosa* PA14 (C)** **monospecies *S. aureus* HG001, (D) monospecies *S. aureus* Newman, and (E-H) dual-species biofilms *P. aeruginosa-S. aureus*.** *P. aeruginosa* and/or *S. aureus* strains were (seeded at 1 × 10^7^ CFU/mL) were grown on hydroxyapatite discs submerged in DFG for 72 hours. CFUs were enumerated per disc before treatment with 32 µg/mL TM18 and after 24 h of TM18 treatment.
